# Effects of Chinese Cabbage Juice Marination Pretreatment on the Quality and Microbial Succession of Fermented Tilapia Fillets

**DOI:** 10.3390/foods15213807

**Published:** 2026-10-23

**Authors:** Zhe Jia, Man Fu, Amani Mohedein Mohammed Ahmed, Shiyuan Dong

**Affiliations:** 1 College of Food Science and Engineering, Ocean University of China, Qingdao 266003, China; jzjzjjj@126.com (Z.J.); fy2024vvv@163.com (M.F.); amanyalgory@yahoo.com (A.M.M.A.); 2 Laboratory of Beibu Gulf Marine Product Healthy Aquaculture and Green Processing, College of Food Engineering, Beibu Gulf University, Qinzhou 535011, China

**Keywords:** tilapia, spontaneously fermented Chinese cabbage juice, fermentation, texture, flavor, bacterial community, microbial metabolic network

## Abstract

The quality improvement of aquatic raw materials through fermentation assisted by fruit- and vegetable-derived ingredients has attracted increasing attention in aquatic food processing. This study investigated the interactions between spontaneously fermented Chinese cabbage juice (SFCCJ) and tilapia fillets during marination pretreatment and characterized changes in physicochemical properties, flavor compounds, and microbial communities of the fillets during subsequent fermentation. SFCCJ pretreatment enriched fermentable substrates (reducing and total sugars) and functional components (phenolics, flavonoids, and nitrate) in tilapia fillets, reduced inter-sample variability in the initial microbial community, and increased the abundances of *Pantoea*, *Weissella*, and *Lactobacillus*. During subsequent fermentation, L* and a* values increased, while hardness, chewiness, and shear force decreased by up to 19.78%, 73.12%, and 44.02%, respectively, and springiness increased by 25.35%. Meanwhile, FAAs, IMP, AMP, lactic and acetic acids accumulated, accompanied by increases in characteristic volatiles such as linalool, 1-heptanol, benzaldehyde, E-2-hexenal, 3-octanone, and 2-hexanone. In contrast, fishy odor-related compounds, including 2-ethyl-1-hexanol, E-2-octenal, and diallyl disulfide, decreased. LAB, particularly *Weissella*, *Lactobacillus*, and *Lactococcus*, dominated the microbial community from 12 h onward. Microbial metabolic network analysis further showed that these genera were closely associated with physicochemical changes and flavor development, whereas *Pantoea*, *Enterobacter*, and *Citrobacter* were associated with biogenic amine accumulation. These findings provide new insights into the targeted quality improvement of tilapia.

## 1. Introduction

Tilapia is one of the major freshwater aquaculture species worldwide due to its high reproductive capacity, broad feeding habits, and tolerance of relatively high stocking densities [1]. According to the “China Fishery Statistical Yearbook” [2], the tilapia aquaculture production in China reached 1.94 million tonnes in 2025. Its rich nutritional profile and low number of intermuscular bones make tilapia a promising raw material for prepared dishes and other value-added products [3]. However, the further utilization of fresh tilapia is constrained by undesirable quality attributes, particularly those related to texture and flavor. Compared with other freshwater fish, fresh tilapia generally exhibits greater hardness and lower springiness. This textural characteristic may be associated with its low fat content and muscle fiber architecture, particularly fiber diameter and density [3,4]. In addition, transportation and temporary holding stress may further impair textural quality by increasing drip and cooking losses [5]. Tilapia also exhibits a pronounced earthy–musty odor that is closely associated with the aquatic environment and microbial metabolites [6]. Geosmin (GSM) and 2-methylisoborneol (2-MIB), mainly produced by cyanobacteria and actinomycetes in culture water, are recognized as the principal contributors to these off-flavors and can accumulate in fish tissues [7]. Pimolrat et al. [8] reported that the intensity of this off-flavor has been closely associated with GSM and 2-MIB concentrations in tilapia.

Fermentation is widely used in the processing of fish such as stinky mandarin fish [9], Mei yu [10], and Suanzuoyu [11] in China. However, most studies have focused on quality improvement in traditional fermented fish products, while the use of fermentation to improve the inherent quality attributes of fish remains limited [1]. Fermentation improves the texture and flavor of fish through interactions between fermentation substrates and microorganisms. During fermentation, microbial carbohydrate metabolism generates organic acids [12], thereby creating conditions that influence protein denaturation, aggregation, and gelation [13]. Meanwhile, endogenous and microbial proteases contribute to protein degradation, releasing peptides and FAAs that may influence the textural properties of fish flesh while serving as important taste compounds and flavor precursors [14]. Furthermore, lipolysis, lipid oxidation, and microbial amino acid metabolism contribute to the formation of volatile flavor compounds, including alcohols, aldehydes, ketones, acids, and esters [15].

Fruit- and vegetable-derived ingredients are rich in carbohydrates, flavor precursors, nitrates, and other functional components and harbor diverse indigenous microorganisms, making them promising adjuncts for food fermentation. Skwarek et al. [16] reported that the addition of freeze-dried tomato pomace to fermented pork sausages significantly increased the total concentrations of alcohols, aldehydes, and acids. Aung et al. [17] found that pineapple and fig powders promoted protein and lipid degradation and improved tenderness in fermented lamb sausages, thereby enhancing their texture and flavor. Chinese cabbage contains fermentable carbohydrates and bioactive compounds, including phenolics and nitrate [18]. It is also widely used in traditional fermented vegetable products, such as kimchi, in which indigenous LAB, particularly *Leuconostoc*, *Weissella*, and *Lactobacillus*, play important roles in fermentation and flavor development [19]. Furthermore, Park et al. [20] reported that kimchi or freeze-dried kimchi powder introduced LAB that rapidly adapted to and proliferated in fermented pork sausages, suppressing Enterobacteriaceae and *Staphylococcus aureus*. Collectively, these studies suggest that fermented Chinese cabbage-derived ingredients may serve as both sources of fermentable substrates and carriers of potentially functional microorganisms. However, the potential of this approach for improving fish quality remains insufficiently explored. Based on these findings, we hypothesized that marination pretreatment of tilapia fillets with fermented Chinese cabbage juice may establish favorable initial conditions for subsequent fermentation, thereby contributing to improvements in texture and flavor. This approach differs from conventional LAB-assisted fish fermentation systems that rely on the inoculation of selected starter cultures, as it may simultaneously modify the initial substrate composition, including sugars and phenolic compounds, and introduce indigenous microorganisms carried by the fermented Chinese cabbage juice.

To test this hypothesis, spontaneously fermented Chinese cabbage juice (SFCCJ) was used for marination pretreatment of tilapia fillets, and changes in their initial substrate composition and microbial community were investigated. Subsequently, sensory attributes, physicochemical properties, flavor compounds, and microbial succession were characterized during fermentation. Furthermore, a Microbial metabolic network was constructed to explore the associations between key microbial genera and changes in physicochemical properties and flavor compounds. This study aimed to evaluate the potential of SFCCJ marination pretreatment as an alternative approach to conventional LAB inoculation for modifying the initial substrate and microbial characteristics of tilapia fillets, and to explore their associations with quality development during subsequent fermentation.

## 2. Materials and Methods

### 2.1. Materials and Chemicals

Live tilapia (*Oreochromis niloticus*; 750–1000 g each) were obtained from Qingdao Tilapia Breeding Farm (Qingdao, Shandong, China) between September and December 2025. After weighing, the fish were placed in oxygenated plastic bags containing chilled water, which were then placed in insulated foam boxes and transported to the laboratory. Upon arrival, the fish were immediately stunned and euthanized by a percussive blow to the head, followed by processing under chilled conditions using a mixture of ice and water.

Chinese cabbage (3000–5000 g each) was harvested in the Jiaodong region of Shandong Province, China, between September and December 2025 and purchased from Jiajiayue Supermarket (No. 456 Haiya Road, Huangdao District, Qingdao, Shandong, China) during the same period. The cabbage was processed on the day of purchase without prolonged storage.

Standards of gallic acid, rutin, nitrate, 5′-nucleotides, and organic acids were purchased from Shanghai Yuanye Biotechnology Co. (Shanghai, China), biogenic amine standards were obtained from Shanghai Macklin Biochemical Technology Co. (Shanghai, China), and C4-C9 *n*-ketones were purchased from Sinopharm Chemical Reagent Co. (Beijing, China). All other chemicals and reagents were of analytical grade.

### 2.2. Preparation of Samples

#### 2.2.1. Preparation of SFCCJ

Fresh Chinese cabbage was juiced using a juice extractor with residue separation. Sodium chloride was added to the juice at 2% (*m*/*m*) and mixed uniformly. The soluble solids content was measured using a portable refractometer and adjusted to Brix 7.0 ± 0.2% by dilution with a 2% NaCl solution. The juice was then aliquoted into boiled–sterilized 500 mL glass jars equipped with one-way exhaust valves and filled nearly to the rim. After being tightly sealed, the juice was spontaneously fermented at 30 °C for 0, 6, or 12 h.

#### 2.2.2. Preparation of Fermented Tilapia Fillets

Fresh tilapia were washed with cold water (4 °C) to remove surface mucus, and then descaled, decapitated, skinned, and eviscerated. The intact dorsal and abdominal muscles were separated, rinsed with cold water, blotted dry, and cut into fillets measuring 3 × 3 × 1 cm (length × width × height). The fillets were mixed uniformly with 2% (*w*/*w*) salt and cured at 4 °C for 30 min. Subsequently, the fillets were pretreated by marination with SFCCJ at a fish-to-marinade ratio of 1:1 (*m*/*m*) at 4 °C for 0, 10, 20, 30, 40, or 50 min. For the control group, SFCCJ was replaced with a 2% (*w*/*w*) NaCl solution. After marination, the fillets were transferred into identically pretreated glass jars, filled nearly to the rim, tightly sealed, and fermented at 20 °C for 0, 6, 12, 18, 24, and 30 h.

### 2.3. Sensory Analysis

Ten assessors, selected and trained with reference to ISO 8586:2023 [21], conducted sensory evaluations of fermented tilapia fillets. Before formal evaluation, the assessors completed three 20 min training sessions using reference samples to familiarize themselves with the sensory attributes and scoring criteria and to promote consistency in scoring. For each fermentation time point, two sets of tilapia fillet samples were prepared. One set was placed on porcelain plates, wrapped in plastic film, and steamed over boiling water for 10 min, while the other set was evaluated raw without further treatment. All ten assessors evaluated both raw and steamed samples at each fermentation time point. The final scores for color, aroma, and texture were based on the combined assessment of raw and steamed samples, while taste was evaluated exclusively using steamed samples. Overall acceptability was determined by considering the sensory characteristics of both raw and steamed samples and the individual attribute scores. All attributes were evaluated using a nine-point scale, with higher scores indicating greater sensory acceptability. Sensory evaluation was conducted across three independent experimental batches. In each batch, each assessor independently assigned one score for each sensory attribute at each fermentation time point. The mean score for each attribute was calculated from 30 individual ratings obtained across the three batches (10 assessors × 3 batches). The detailed scoring criteria are presented in Table S1.

### 2.4. Texture Profile Analysis (TPA) and Shear Force Evaluation

TPA and shear force were measured using a texture analyzer (TMS-Pro, Food Technology Corporation, Sterling, VA, USA). Tilapia fillets were cut into 1.5 × 1 × 1 cm cubes from identical anatomical regions. TPA was performed according to Zhao et al. [1]. Samples were compressed to 50% of their original thickness at 2 mm/s with a trigger force of 0.05 N and an initial probe-to-base distance of 20 mm. Hardness, springiness, and chewiness were obtained from the force–time curves. Shear force was measured according to Li et al. [22] with slight modifications. Samples were sheared parallel to the myofibril orientation using a BSK probe, with pre-test and post-test speeds of 2.0 and 10.0 mm/s, respectively, and a test distance of 50 mm.

### 2.5. Color Measurement

The color of tilapia fillets was measured using a chroma meter (CR-400; Konica Minolta, Tokyo, Japan) based on the CIE color system and expressed as L^*^, a^*^, and b^*^. The total color difference (△E) was calculated according to Yue et al. [23] as follows:

(1) $$△E=\sqrt{{\left(L^{*}-L_{0}^{*}\right)}^{2}+{\left(a^{*}-a_{0}^{*}\right)}^{2}+{\left(b^{*}-b_{0}^{*}\right)}^{2}}$$

where L_0_^*^, a_0_^*^, and b_0_^*^ represent the color parameters of the initial tilapia fillets, and L^*^, a^*^, and b^*^ represent those of samples fermented for different durations.

### 2.6. pH Measurement and Microbial Enumeration

The pH was determined by homogenizing 5.0 g of minced fillet with 45 mL of distilled water, followed by determination using a digital pH meter (IS128; Yimai Shanghai, Shanghai, China). For microbial enumeration, 3.0 g of sample was homogenized with 27 mL of sterile 0.85% NaCl solution. Serial dilutions were plated on plate count agar (PCA), MRS agar, and violet red bile glucose agar (VRBGA) for enumeration of total viable counts (TVC), lactic acid bacteria (LAB), and Enterobacteriaceae, respectively. PCA and MRS plates were incubated at 37 °C for 48 h, VRBGA plates were incubated at 37 °C for 24 h.

### 2.7. Analysis of Substrate Components

Total phenolic and flavonoid contents were determined using the Folin–Ciocalteu and aluminum nitrate colorimetric methods, respectively. For total phenolics, 5.0 g of sample was extracted with 10 mL of 80% ethanol and centrifuged at 5000 rpm for 5 min at 4 °C. The supernatant was reacted with 7% Na_2_CO_3_ and distilled water for 90 min in the dark, and absorbance was measured at 760 nm. For total flavonoids, 5.0 g of sample was extracted with 5 mL of 30% ethanol and centrifuged under the same conditions. The supernatant was sequentially reacted with 5% NaNO_2_, 10% Al(NO_3_)_3_, and 10% NaOH, diluted to 25 mL with 30% ethanol, and measured at 510 nm after 15 min. Total phenolic and flavonoid contents were quantified using gallic acid and rutin standard curves, respectively.

Reducing sugar and total sugar contents were determined using the 3,5-dinitrosalicylic acid method and expressed as mg/g. Nitrate content was determined according to Andrade [24] with slight modifications. A 5.0 g sample was extracted with 50 mL of distilled water, 2.5 mL of ammonia buffer (pH 9.6–9.7), and 1.0 g of activated carbon for 30 min. After clarification with 1 mL each of 150 mg/mL K_4_[Fe(CN)_6_] and 300 mg/mL ZnSO_4_ solutions, the extract was filtered and appropriately diluted. Absorbance was measured at 219 nm, and nitrate content was quantified using a nitrate standard curve. Ascorbic acid and citric acid contents were determined using commercial assay kits (A009-1-1 and A128-1-1, Nanjing Jiancheng Bioengineering Institute Co., Ltd., Nanjing, China), respectively, and expressed as μg/g. All measured substrate component contents were expressed on a wet weight basis.

### 2.8. Determination of Biogenic Amines (BAs)

BAs were extracted by homogenizing 5.0 g of minced fillet with 20 mL of 5% trichloroacetic acid, followed by centrifugation at 8000 rpm for 20 min at 4 °C. The supernatant was derivatized with dansyl chloride and analyzed using an HPLC system (Agilent 1260 Infinity, Agilent Technologies, Inc., Santa Clara, CA, USA) equipped with a ZORBAX Eclipse Plus C18 column (250 mm × 4.6 mm, 5 μm). Gradient elution with ultrapure water and acetonitrile was performed according to Zhao et al. [1] at a flow rate of 1 mL/min. The column temperature was maintained at 40 °C, and 20 μL of derivatized sample was injected and detected at 254 nm using a UV detector. BAs were quantified using a standard curve constructed from peak area versus standard concentration (1–100 mg/L).

### 2.9. Analysis of Taste-Related Compounds

Total free amino acids (T-AA) were determined using a commercial assay kit (A026-1-1, Nanjing Jiancheng Bioengineering Institute Co., Ltd., Nanjing, China) and expressed as mg/kg.

5′-Nucleotides were determined according to Qiu et al. [25] with slight modifications. Minced fillet (5.0 g) was homogenized with 20 mL of prechilled 10% (*v*/*v*) perchloric acid at 10,000 r/min for 2 min and centrifuged at 10,000 rpm and 4 °C for 15 min. The supernatant was filtered through a 0.22 μm membrane and analyzed by HPLC using a reversed-phase ZORBAX Eclipse Plus C18 column (250 mm × 4.6 mm, 5 μm). The mobile phase consisted of acetonitrile and 10 mmol/L KH_2_PO_4_ containing 1.45 mmol/L tetrabutylammonium hydrogen sulfate (pH 4.0) at a ratio of 3:97 (*v*/*v*). The column temperature, flow rate, and detection wavelength were 30 °C, 1 mL/min, and 254 nm, respectively. Quantification was performed using calibration curves of 5′-CMP, 5′-UMP, 5′-GMP, 5′-IMP, and 5′-AMP.

Organic acids were determined according to Zhang et al. [26] with slight modifications. Minced fillet (5.0 g) was homogenized with 25 mL of distilled water for 2 min and centrifuged at 10,000 rpm for 15 min. The supernatant was filtered through a 0.22 μm membrane and analyzed by HPLC using a reversed-phase ZORBAX Eclipse Plus C18 column (250 mm × 4.6 mm, 5 μm). The mobile phase was 20 mmol/L NaH_2_PO_4_ solution adjusted to pH 2.7. The column temperature, flow rate, and detection wavelength were 35 °C, 0.9 mL/min, and 210 nm, respectively. Organic acids were quantified using calibration curves of oxalic, tartaric, lactic, acetic, citric, and succinic acids.

### 2.10. Analysis of Volatile Compounds by GC-IMS

Volatile compounds were analyzed by GC-IMS according to Li et al. [27] with slight modifications. Minced fillet (2.0 g) was placed in a 20 mL headspace vial and incubated at 60 °C for 20 min with agitation at 500 rpm. Headspace samples (500 μL) were automatically injected using a CTC-PAL autosampler (CTC Analytics AG, Zwingen, Switzerland) with the syringe maintained at 65 °C. Separation was performed on an MXT-WAX column (15 m × 0.53 mm ID; G.A.S., Dortmund, Germany) at 60 °C using high-purity nitrogen (99.99%) as the carrier gas. The carrier-gas flow rate was programmed at 2 mL/min initially, 5 mL/min at 2 min, 10 mL/min at 10 min, 100 mL/min at 20 min, and 150 mL/min at 25 min. The drift tube was maintained at 45 °C, with nitrogen as the drift gas at 150 mL/min. The syringe was flushed with nitrogen for 30 s before and 5 min after each analysis to prevent cross-contamination. C4-C9 *n*-ketones were used as reference compounds to calculate retention indices (RIs). The corresponding RI, retention times (Rt), and relative drift times (Dt) of the detected volatile compounds are provided in Table S5. Volatile compounds were tentatively identified by matching their GC-IMS signals against the GC×IMS Library Search database and the NIST 14 library using Laboratory Analytical Viewer (LAV) software (v1.0.3). No internal standard was used, and the GC-IMS peak intensities were normalized using LAV software and expressed as relative peak intensities for semi-quantitative comparison. All analyses were performed in triplicate.

### 2.11. Microbial Community Analysis

A 5.0 g sample was homogenized with 45 mL of sterile 0.85% NaCl solution and centrifuged at 12,000 rpm for 10 min at 4 °C. The microbial pellet was collected for genomic DNA extraction and 16S rRNA gene sequencing according to Li et al. [28]. For tilapia fillet samples, the V3-V4 region was amplified using primers 341F (5′-CCTAYGGGRBGCASCAG-3′) and 806R (5′-GGACTACHVGGGTWTCTAAT-3′), whereas the V5-V7 region of Chinese cabbage juice samples was amplified using primers 799F (5′-AACMGGATTAGATACCCKG-3′) and 1193R (5′-ACGTCATCCCCACCTTCC-3′). Purified amplicons were sequenced on an Illumina MiSeq PE300 platform. Raw reads were demultiplexed, quality-filtered using fastp (v0.19.6), and merged using FLASH (v1.2.11). Operational taxonomic units (OTUs) were clustered at 97% sequence similarity using UPARSE (v7.0.1090), and taxonomic assignment was performed using the RDP Classifier (v2.11). Non-bacterial sequences assigned to chloroplasts and mitochondria were excluded prior to downstream microbial community analysis.

### 2.12. Statistical Analysis

All experimental assays were carried out in triplicate, with the resulting data presented as the mean ± standard deviation (SD). Differences among fermentation times within each group were analyzed using one-way analysis of variance (ANOVA) followed by Duncan’s multiple range test. Differences between the SFCCJ and control groups at the same fermentation time were assessed using independent-samples *t*-tests, with *p* < 0.05 considered statistically significant. Principal component analysis (PCA) and partial least squares discriminant analysis (PLS-DA) were performed using OmicStudio tools (https://www.omicstudio.cn/tool, accessed on 22 June 2026). Pearson correlation analysis was conducted in SPSS (v23.0) to assess relationships between microbial communities and physicochemical properties or volatile compounds. The Benjamini–Hochberg false discovery rate (FDR) procedure was applied to the *p*-values obtained from differential analysis of volatile compounds and Pearson correlation analyses, with adjusted *p* < 0.05 considered significant. Correlation networks were generated using Cytoscape v3.10.3.

## 3. Results and Discussion

### 3.1. Effects of Chinese Cabbage Juice Fermentation Duration on the Quality of Fermented Tilapia Fillets

According to the LAB growth curve in SFCCJ (Figure S1), fresh juice (lag phase), 6 h fermented SFCCJ (exponential phase), and 12 h fermented SFCCJ (stationary phase) were selected for tilapia fillet pretreatment. The textural properties, pH, LAB counts, and Enterobacteriaceae counts of tilapia fillets after 18 h of fermentation are presented in Table S2. Compared with fresh tilapia fillets (23.52 N), the 2% NaCl solution control exhibited significantly higher hardness (25.27 N). Among the three cabbage juice pretreatments (0, 6, and 12 h), the 6 h fermented SFCCJ group exhibited the lowest hardness (21.27 vs. 23.36 and 23.36 N, *p* < 0.05), chewiness (0.48 N.mm vs. 0.67 and 0.68 N.mm, *p* < 0.05), and shear force (5.64 N), together with the highest springiness (3.61 vs. 3.31 and 3.01 mm, *p* < 0.05), indicating more favorable textural characteristics.

As the fermentation duration of Chinese cabbage juice increased from 0 to 6 and 12 h, the pH of fermented tilapia fillets decreased significantly from 6.30 to 5.82 and 5.48 (*p* < 0.05), respectively, whereas LAB counts increased from 6.42 to 7.07 and 7.98 log CFU/g (*p* < 0.05), respectively. Compared with LAB in fresh juice (lag phase), those in 6 h fermented SFCCJ were in the exponential growth phase, potentially exhibiting greater adaptability and facilitating subsequent fermentation [29], which may partly explain the superior textural properties of the 6 h group. Despite its higher LAB count, the greater acidification associated with 12 h fermented SFCCJ may have limited further textural improvement. Consistently, Latorre-Moratalla et al. [30] reported that fermented fish sausages exhibited significantly increased hardness and chewiness but decreased springiness when the pH value was below 5.4. Furthermore, Enterobacteriaceae counts were comparable between the fresh juice and 6 h groups (4.79 and 4.74 log CFU/g, respectively) but significantly higher in the 12 h group (5.60 log CFU/g). Collectively, 6 h fermented SFCCJ provided the most favorable balance of textural properties, acidification, and microbial characteristics and was therefore selected for subsequent experiments.

### 3.2. Effects of SFCCJ Pretreatment on the Initial Substrate and Microbial Characteristics of Tilapia Fillets

#### 3.2.1. Key Substrate Components and Microbial Counts

Tilapia fillets were pretreated with 6 h fermented SFCCJ for 0–50 min, and the changes in key substrate components and microbial counts of tilapia fillets during marination were investigated (Figure S2). After 10 min of marination, the total phenolic and flavonoid contents in tilapia fillets increased significantly from 0.149 to 0.179 mg/g (20.13%) and from 30.52 to 59.39 μg/g (94.59%), respectively. The increased phenolic compounds may serve as important bioactive components during subsequent fermentation due to their antioxidant and antimicrobial activities [31]. At 30 min of marination, reducing sugar and total sugar contents increased significantly from 4.74 to 5.50 mg/g (16.03%) and from 4.03 to 6.30 mg/g (56.33%), respectively. Meanwhile, nitrate content and LAB counts peaked at 30 min, increasing from 0.40 to 1.18 mg/g (195.00%) and from 4.16 to 4.53 log CFU/g, respectively (*p* < 0.05). As essential carbon sources for fermentative microorganisms, reducing sugars and total sugars provide available substrates and energy for microbial growth and metabolism, thereby providing favorable substrates for subsequent fermentation [32]. However, extending the marination time to 50 min resulted in a significant reduction in total flavonoid content to the initial level, possibly due to oxidation or interactions with food matrix components, which may affect flavonoid stability and extractability [33]. Moreover, TVC and LAB counts declined below their initial levels, possibly reflecting microbial redistribution between the fish tissue and the SFCCJ marination solution during prolonged marination. Significant decreases in ascorbic acid and citric acid contents were also observed. Collectively, these findings suggest that 30 min of SFCCJ marination provided a favorable balance between the enrichment of fermentable substrates and functional components and the maintenance of microbial counts, whereas prolonged marination diminished these benefits. Therefore, 30 min was selected as the optimal marination duration among the conditions tested for subsequent fermentation.

#### 3.2.2. Microbial Community Composition

High-throughput sequencing was used to characterize the microbial communities of SFCCJ, fresh tilapia fillets (BM), and tilapia fillets pretreated with SFCCJ for 30 min (AM). PCA (Figure S3) explained 93.28% of the total variance (PC1: 86.71%, PC2: 6.57%) and revealed a clear separation between BM and AM along PC1, indicating that SFCCJ pretreatment markedly altered the initial microbial community composition of tilapia fillets. The tighter clustering of AM replicates further suggested reduced microbial variability and greater initial consistency for subsequent fermentation. At the phylum level (Table S3), the microbial community of fresh tilapia fillets was dominated by *Cyanobacteria* (60.01%), *Proteobacteria* (25.37%), *Actinobacteriota* (9.56%), and *Firmicutes* (2.79%). After SFCCJ pretreatment, the abundances of *Cyanobacteria* and *Actinobacteriota* decreased to 52.79% and 3.39%, respectively (*p* < 0.01), whereas those of *Proteobacteria* and *Firmicutes* increased to 36.58% and 5.45%, respectively (*p* < 0.01). Consistent with previous studies, *Proteobacteria* and *Firmicutes* have been identified as predominant bacterial phyla during tilapia surimi fermentation [1]. In contrast, the high abundance of *Cyanobacteria* in fresh tilapia fillets may be associated with the aquaculture environment and dietary sources [34].

Further analysis at the genus level (Table S4) showed that SFCCJ pretreatment significantly reduced the relative abundances of *Plectonema*, *Chroococcus*, *Renibacterium*, *Pseudomonas*, and *Stenotrophomonas* in tilapia fillets (*p* < 0.01). Conversely, the relative abundances of *Pantoea*, *Weissella*, and *Lactobacillus* increased significantly from 0.08%, 0.19%, and 0.02% to 1.25%, 1.35%, and 1.18%, respectively (*p* < 0.01). Notably, these genera were also present in SFCCJ at relatively high abundances (42.74%, 6.95%, and 6.66%, respectively), suggesting that SFCCJ may have served as an important external source contributing to their enrichment in tilapia fillets. Similarly, Song et al. [35] demonstrated that vegetable-derived microorganisms influence microbial community assembly and metabolic profiles during kimchi fermentation, suggesting that microorganisms from SFCCJ may similarly contribute to the initial microbial community of tilapia fillets and influence subsequent fermentation. Previous studies have identified *Pantoea* as a dominant genus during the fermentation of Meiyu [10] and fish sauce [36], with its growth positively associated with desirable flavor development in fish sauce fermentation. Moreover, *Weissella* and *Lactobacillus* are important fermentation-associated genera involved in carbohydrate fermentation, organic acid production, and flavor development [37,38]. Overall, SFCCJ pretreatment simultaneously enriched fermentable substrates and potentially functional microorganisms in tilapia fillets. This approach differs from conventional LAB-assisted fermentation systems that rely primarily on the inoculation of selected starter cultures and may establish favorable conditions for subsequent fermentation.

### 3.3. Quality Changes in SFCCJ-Pretreated Tilapia Fillets During Fermentation

#### 3.3.1. Sensory Evaluation

**Figure 1:**
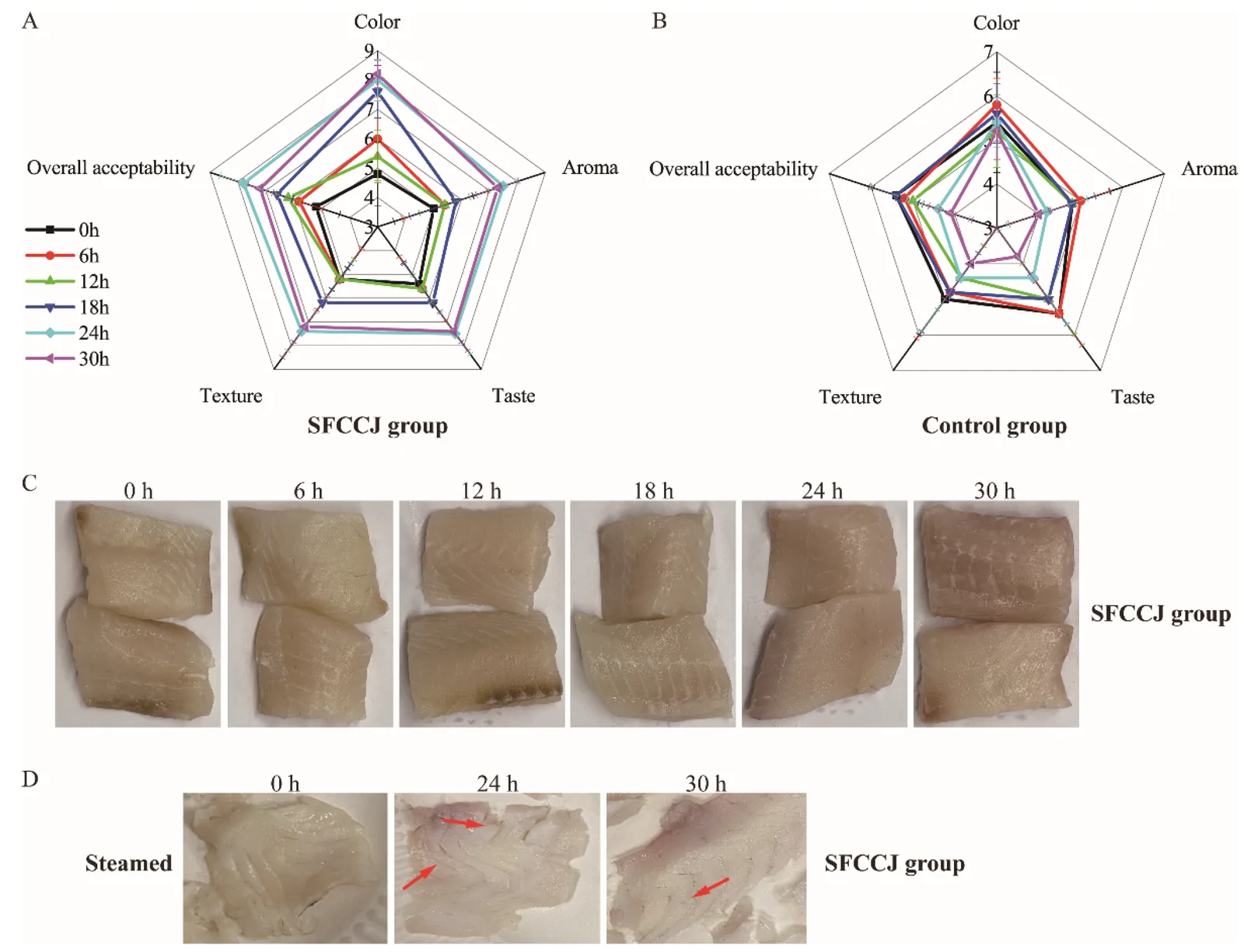
Sensory scores and appearance of tilapia fillets during fermentation. (**A**) Sensory scores of the SFCCJ group. (**B**) Sensory scores of the control group. (**C**,**D**) Appearance of raw and steamed tilapia fillets in the SFCCJ group at different fermentation times, respectively. The red arrows in (**D**) indicate the garlic-clove-like structure formed by the partial separation of muscle tissues after steaming.

Sensory evaluation showed a marked improvement in the sensory quality of SFCCJ-pretreated tilapia fillets during fermentation (Figure 1A). From 0 to 24 h, the scores for aroma, taste, texture, and overall acceptability increased significantly from 5.00, 5.40, 5.20, and 5.20 to their respective maxima of 7.50, 7.50, 7.40, and 7.80 (*p* < 0.05). These scores were significantly higher than those of the control group from 12 h onward (Figure 1B; *p* < 0.05). The color score increased from 4.80 initially to 8.00 at 24 h and reached a maximum of 8.20 at 30 h, remaining significantly higher than that of the control group from 18 h onward (*p* < 0.05). Notably, the fermented fillets developed a distinctive fresh vegetable-like aroma accompanied by reduced fishy odor and enhanced umami, representing a characteristic sensory feature associated with SFCCJ pretreatment. These sensory improvements may be associated with changes in volatile flavor compounds and the accumulation of umami-related substances during fermentation, potentially influenced by microbial metabolism [1]. Although the color score continued to increase, overall acceptability decreased to 7.20 at 30 h (*p* > 0.05), potentially due to increased sourness.

Changes in appearance further supported the sensory improvement (Figure 1C). As fermentation progressed, the fillet surface became increasingly glossy, and a distinct pinkish sheen developed after 24 h. These changes became more evident after steaming (Figure 1D). The 0 h samples showed a dull, uneven yellowish appearance and a relatively dry surface, whereas the 24 and 30 h samples exhibited a bright white-to-pink color with clearly defined muscle layers. Partial separation of muscle tissues appeared at 24 h and became more pronounced at 30 h, resulting in a garlic-clove-like structure. This structural development may be associated with moderate proteolysis that weakens muscle tissue connections, facilitating tissue separation, while fermentation-induced changes in protein gel-forming properties may help maintain the integrity and elasticity of the separated muscle segments [9]. Notably, this garlic-clove-like structure developed within a relatively short fermentation period and was accompanied by improved tenderness and springiness. In contrast, fermented fish with similar tissue separation, such as stinky mandarin fish, generally require several days of fermentation and are often associated with increased hardness [9].

#### 3.3.2. Physicochemical Properties

The changes in textural properties, color, pH, TVC and LAB of the SFCCJ and control groups during fermentation are shown in Table 1. In the SFCCJ group, hardness, chewiness, and shear force generally decreased, whereas springiness increased, with the most pronounced textural improvement observed at 24 h. Compared with the initial samples, hardness, chewiness, and shear force decreased by 19.78%, 73.12%, and 44.02%, respectively, while springiness increased by 25.35% (*p* < 0.05). Notably, at 24 h, the SFCCJ group exhibited significantly lower hardness, chewiness, and shear force but higher springiness than the control group (*p* < 0.05). These improvements may be associated with progressive protein degradation and conformational changes in myofibrillar proteins [1,39], contributing to enhanced tenderness and elasticity. At 30 h, springiness in the SFCCJ group decreased significantly, indicating that prolonged fermentation provided no further textural improvement.

The pH of the SFCCJ group decreased from 6.71 to 5.77 within the first 24 h and further declined to 5.42 at 30 h. In contrast, the control group showed a slower pH decline, with a value of 6.58 at 24 h. Meanwhile, TVC in the SFCCJ group increased significantly from 5.75 to 7.37 log CFU/g at 30 h, whereas LAB counts increased significantly from 4.96 to a maximum of 8.74 log CFU/g at 24 h (*p* < 0.05), significantly higher than the corresponding control value of 6.00 log CFU/g. These findings suggest that SFCCJ pretreatment promoted LAB proliferation and acidification during subsequent fermentation, consistent with observations in fermented tilapia surimi [1].

As fermentation progressed, L* and a* increased continuously in the SFCCJ group, whereas b* decreased, indicating increased lightness and redness and reduced yellowness. These changes were consistent with the visual observations in Figure 1C and similar to those reported for stinky mandarin fish fermentation [9]. Compared with the control group, the SFCCJ group exhibited significantly higher L* and a* values at 24 h (*p* < 0.05). The increased lightness and reduced yellowness may be associated with LAB-mediated acidification, which influences protein gelation and light-scattering properties [9], whereas the increased redness may be related to a microbial reduction in nitrate introduced by SFCCJ pretreatment and subsequent formation of nitrosyl pigments [40]. Furthermore, ΔE in the SFCCJ group increased markedly after 12 h, reaching 8.47 and 13.51 at 24 and 30 h (*p* < 0.05), respectively, exceeding the corresponding control values and indicating more pronounced color changes relative to the initial samples.

Most BAs, including phenylethylamine, putrescine, cadaverine, histamine, tyramine, and spermine, increased during fermentation (Table 2), with putrescine, cadaverine, and histamine being predominant. In contrast, spermidine gradually decreased and became undetectable at 18 h in the SFCCJ group and at 30 h in the control group. Notably, total BA content at 24 h was lower in the SFCCJ group (150.46 mg/kg) than in the control group (210.42 mg/kg), suggesting that SFCCJ pretreatment may have limited BA accumulation during fermentation. Histamine and tyramine are considered among the most toxic BAs in fermented foods [41]. At 30 h, their concentrations in the SFCCJ group remained below 20 and 10 mg/kg, respectively, and were lower than those in the control group. Nevertheless, putrescine and cadaverine, commonly associated with fish spoilage, may enhance histamine toxicity and participate in the formation of potentially carcinogenic compounds under suitable conditions [42,43]. Therefore, their accumulation during fermentation warrants attention despite the absence of established safety limits.

Overall, comparison with the control group indicated that SFCCJ pretreatment improved the textural and color properties of tilapia fillets, promoted LAB growth and acidification, and was associated with lower BA accumulation. To further investigate quality development in SFCCJ-pretreated tilapia fillets, the subsequent analyses focused on changes in taste-related compounds, volatile profiles, and microbial communities within the SFCCJ group during fermentation.

#### 3.3.3. Taste Properties

##### Total FAAs

**Figure 2:**
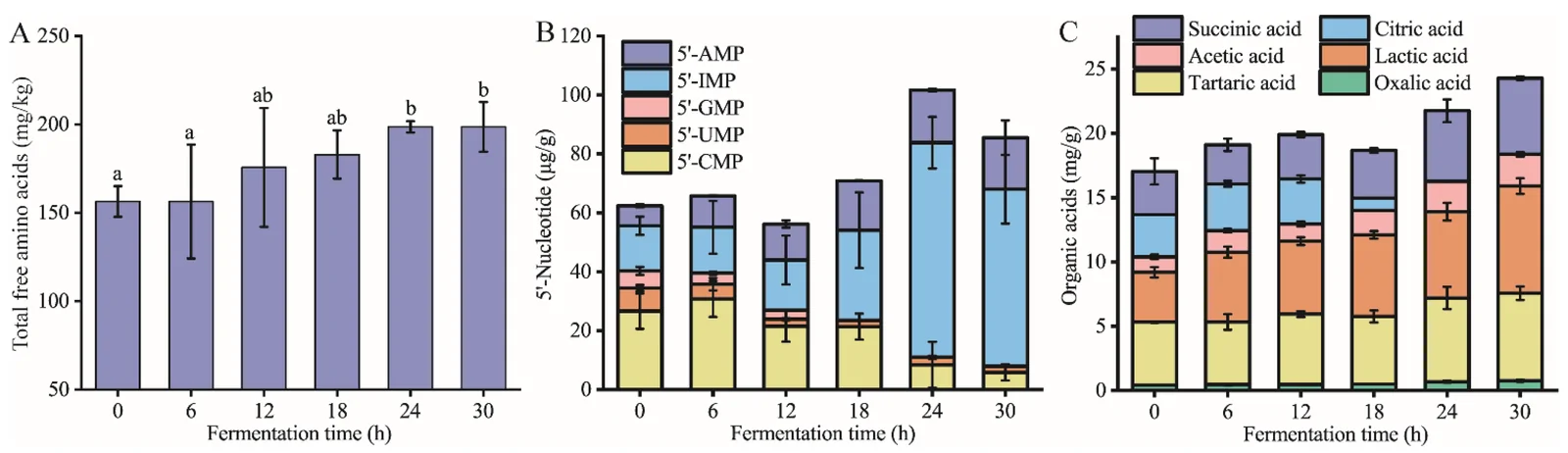
Changes in total free amino acid content (**A**), 5′-nucleotides contents (**B**) and organic acid contents (**C**) during the fermentation of tilapia fillets. Different lowercase letters indicate significant differences among fermentation times (*p* < 0.05).

As shown in Figure 2A, the T-AA content in tilapia fillets increased significantly from 156.37 mg/kg at 0 h to 198.59 mg/kg at 24 h. A similar trend was reported for low-salt fermented tilapia [44]. FAAs contribute directly to taste and can also serve as precursors of volatile compounds [45]. Their accumulation may therefore have contributed to the improved taste and overall flavor.

##### 5′-Nucleotides

5′-Nucleotides are important taste-active compounds contributing to freshness and umami perception [46]. As shown in Figure 2B, the total 5′-nucleotide content in tilapia fillets increased significantly from 62.37 μg/g at 0 h to 101.61 μg/g at 24 h, followed by a decline to 85.81 μg/g at 30 h (*p* < 0.05). This increase was mainly driven by IMP and AMP, whereas CMP, UMP, and GMP generally decreased, with GMP becoming undetectable after 18 h. IMP reached a maximum of 72.83 μg/g at 24 h, while AMP increased significantly at 18 h (*p* < 0.05) and subsequently remained relatively stable at 16.82−17.79 μg/g. In contrast, decreases in IMP and AMP have been commonly observed during fish fermentation [47]. The accumulation of these nucleotides depends on the balance between their formation and degradation through endogenous ATP degradation and subsequent breakdown [48], suggesting that the relatively short fermentation period may have limited its further degradation. Moreover, certain strains of *Weissella* and *Lactobacillus* have been reported to influence nucleic acid hydrolysis and nucleotide-related metabolism during fish fermentation [49]. Thus, the relatively short fermentation period and microbial activity may have contributed to the observed accumulation of IMP and AMP. As IMP and AMP contribute directly to umami and can act synergistically with glutamic and aspartic acids [50,51], their accumulation, particularly at 24 h, likely contributed to the enhanced umami perception observed in the sensory evaluation.

##### Organic Acids

Organic acids are important flavor compounds in fermented foods and also contribute to the inhibition of spoilage and pathogenic microorganisms [12]. As shown in Figure 2C, lactic, tartaric, succinic, acetic, and oxalic acids increased significantly during fermentation (*p* < 0.05), with lactic and acetic acids reaching 832.82 and 246.40 mg/100 g at 30 h, respectively. Their accumulation was consistent with the progressive decrease in pH (Table 1). In contrast, citric acid decreased continuously and became undetectable at 24 h, possibly due to its utilization by LAB [52]. The accumulation of lactic and acetic acids may be associated with carbohydrate metabolism by LAB. *Weissella*, a typically heterofermentative genus, may metabolize glucose through the phosphoketolase pathway, generating glyceraldehyde-3-phosphate and acetyl phosphate as intermediates that can be further converted into lactic and acetic acids, respectively [12]. Meanwhile, certain *Lactobacillus* species may primarily metabolize glucose via the EMP pathway, with L-lactate dehydrogenase catalyzing the conversion of pyruvate to lactic acid [53]. These metabolic activities may have contributed to the observed organic acid accumulation and pH reduction. Lactic and succinic acids can enhance umami synergistically with umami amino acids, whereas excessive acetic acid may contribute to a pungent sour taste [54,55].

#### 3.3.4. Aroma Profile

**Figure 3:**
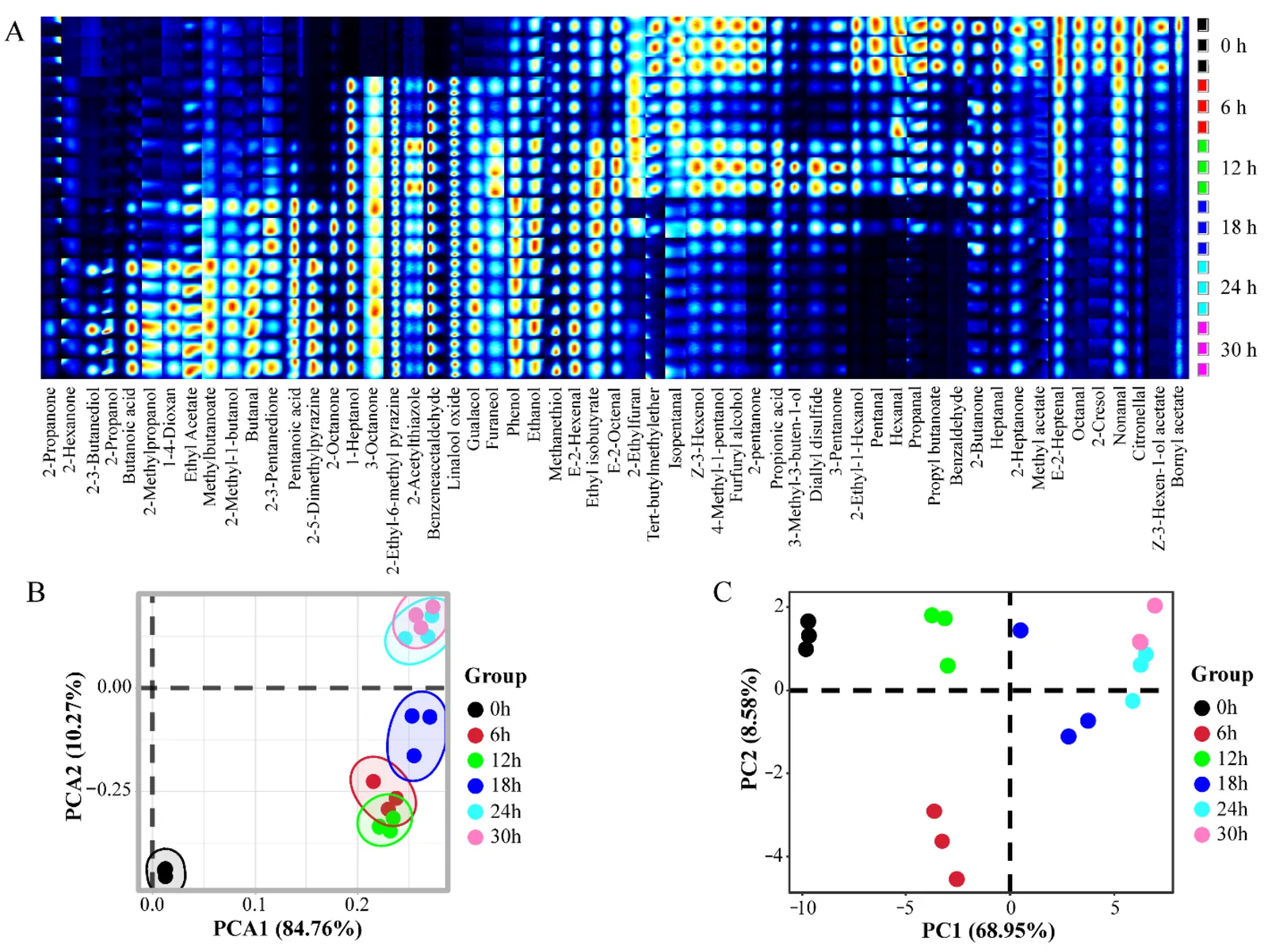
GC-IMS analysis of fermented tilapia fillets. (**A**) Fingerprints of fermented tilapia fillets. (**B**) PCA score plot of GC-IMS data. (**C**) PLS-DA score plot of GC-IMS data.

The aroma profile of tilapia fillets was analyzed by GC–IMS. As shown in Figure 3A and Table S5, the diversity and abundance of volatile compounds generally increased as fermentation progressed. A total of 57 volatile compounds were tentatively identified, including 14 alcohols, 14 aldehydes, 9 ketones, 7 esters, 3 acids, 3 phenols, 2 sulfides, 2 ethers, 2 pyrazines, and 1 furan. Similar increases in volatile diversity have been reported in low-salt fermented fish [15]. PCA and PLS-DA clearly separated samples from different fermentation times, indicating marked changes in volatile profiles during fermentation (Figure 3B,C). Based on VIP > 1 and -log_10_*p* > 3, 24 characteristic volatile compounds were identified as significantly changed (Table S6). These results indicated that fermentation markedly altered the volatile profile of tilapia fillets, with decreases in some initially abundant compounds and increases in other characteristic aroma compounds.

Alcohols, aldehydes, and ketones were the predominant characteristic volatile compounds. Among the alcohols, linalool, 1-heptanol, 2-methylpropanol, and 2-propanol increased (Table S7), contributing floral, green, and alcoholic odors. In contrast, 2-ethyl-1-hexanol, an important contributor to fishy odor in freshwater fish, declined substantially between 18 and 24 h. Linalool, a floral compound commonly associated with plant materials and spices [56], may have been introduced by SFCCJ pretreatment, while relatively high levels of 1-heptanol have also been reported in naturally fermented tilapia surimi [1]. Among the aldehydes, benzaldehyde and E-2-hexenal increased, contributing fruity and green notes, whereas E-2-octenal and phenylacetaldehyde decreased. Aldehyde formation may be associated with lipid oxidation and Strecker degradation of amino acids [57]. Notably, the decrease in E-2-octenal, a key contributor to fishy odor in fresh silver carp [58], may also have contributed to odor improvement. Among the ketones, 3-octanone and 2-hexanone increased markedly, and their herbal and fruity odors may have reduced the perception of fishy odor [59].

Methyl butanoate increased during fermentation, consistent with the contribution of esters to fruity aromas in dry-fermented sausages [60]. Propanoic and butanoic acids also increased significantly and, as LAB-derived short-chain fatty acids, may have served as precursors for ester formation [61]. In addition, 2-methoxyphenol, phenol, and 2-ethyl-6-methylpyrazine increased markedly, contributing pungent, roasted, and nutty odors [62,63]. In contrast, diallyl disulfide, a sulfur compound potentially associated with off-odors [64], decreased significantly after 24 h. Overall, SFCCJ pretreatment appeared to facilitate the accumulation of volatile compounds associated with floral, fruity, green, and roasted notes during tilapia fillet fermentation, while reducing several compounds associated with fishy odors.

### 3.4. Microbial Community Succession in SFCCJ-Pretreated Tilapia Fillets During Fermentation

**Figure 4:**
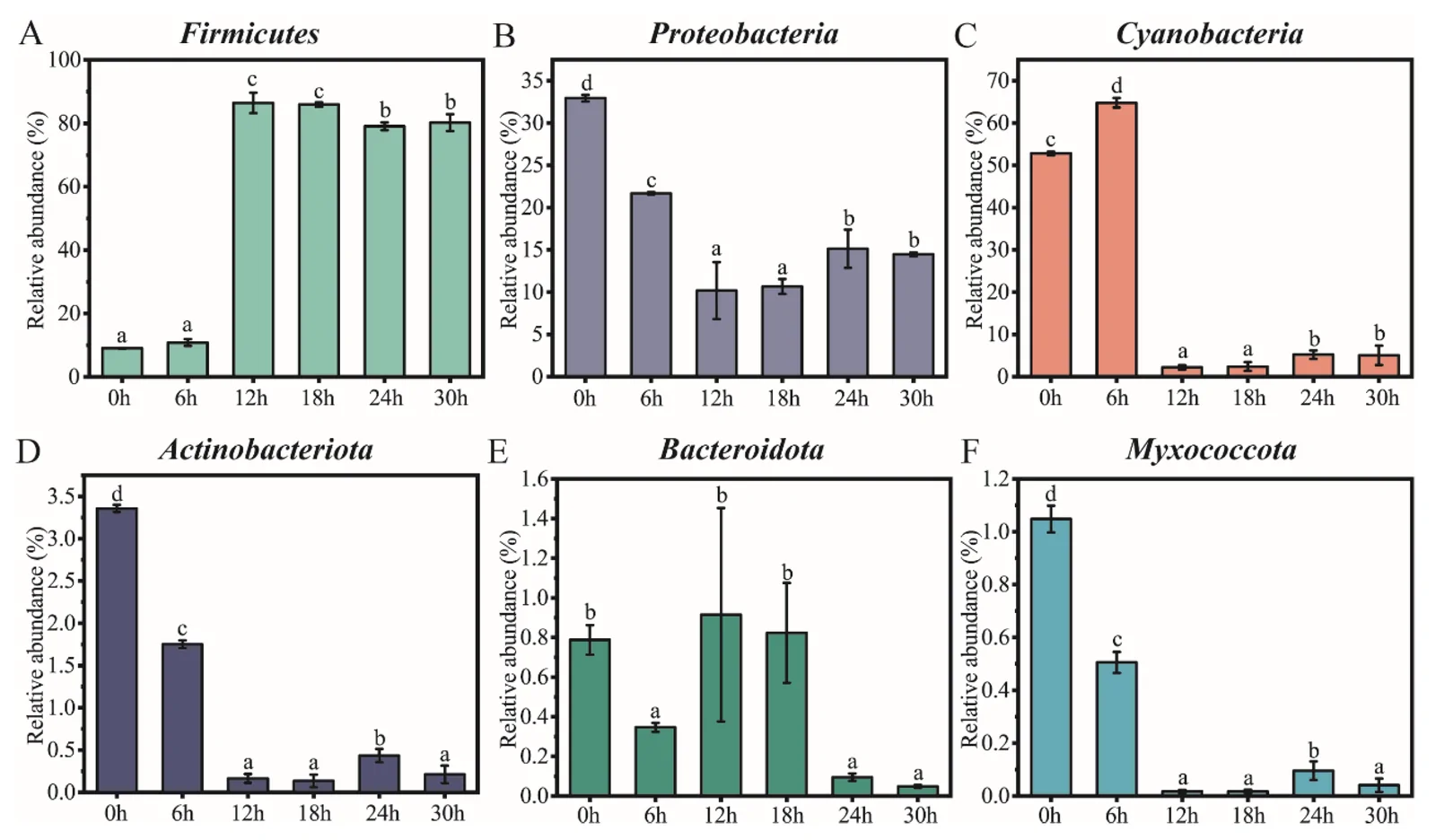
Relative abundances of bacterial phyla in tilapia fillets during fermentation. (**A**) *Firmicutes*; (**B**) *Proteobacteria*; (**C**) *Cyanobacteria*; (**D**) *Actinobacteriota*; (**E**) *Bacteroidota*; (**F**) *Myxococcota*. Different lowercase letters indicate significant differences among fermentation times (*p* < 0.05).

**Figure 5:**
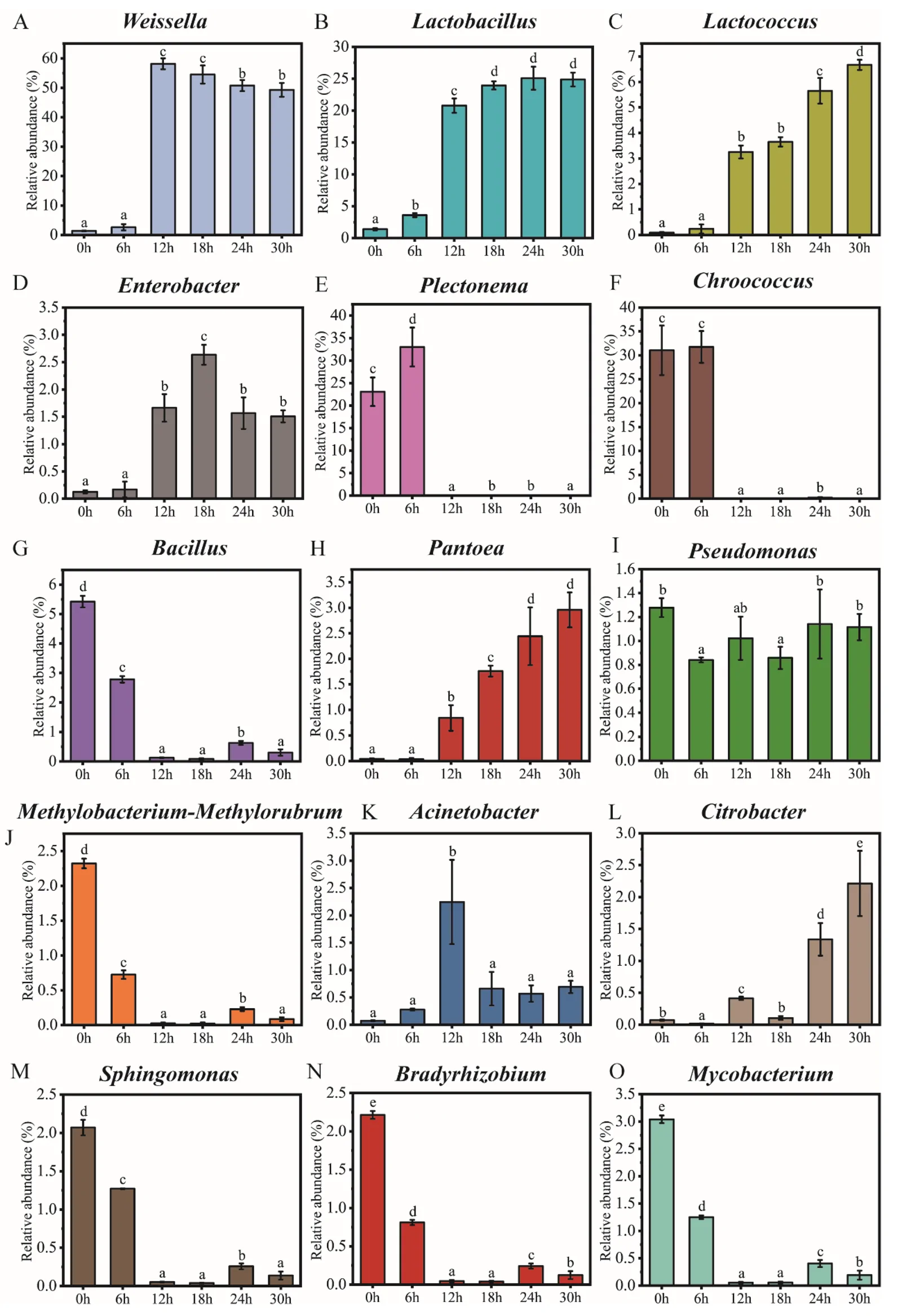
Bacterial genera with a relative abundance greater than 1% in tilapia fillets during fermentation. (**A**) *Weissella*; (**B**) *Lactobacillus*; (**C**) *Lactococcus*; (**D**) *Enterobacter*; (**E**) *Plectonema*; (**F**) *Chroococcus*; (**G**) *Bacillus*; (**H**) *Pantoea*; (**I**) *Pseudomonas*; (**J**) *Methylobacterium*-*Methylorubrum*; (**K**) *Acinetobacter*; (**L**) *Citrobacter*; (**M**) *Sphingomonas*; (**N**) *Bradyrhizobium*; (**O**) *Mycobacterium*. Different lowercase letters indicate significant differences among fermentation times (*p* < 0.05).

The microbial composition of tilapia fillets during fermentation was characterized at both the phylum (Figure 4) and genus (Figure 5) levels. *Cyanobacteria* and *Proteobacteria* were the dominant phyla of tilapia fillets before fermentation, with relative abundances of 52.80% and 32.93%, respectively, followed by *Actinobacteriota* (3.36%) and *Myxococcota* (1.05%). As fermentation progressed, the abundance of *Firmicutes* significantly increased and peaked at 86.45% at 12 h, whereas *Cyanobacteria*, *Proteobacteria*, and *Actinobacteriota* declined substantially. Similar results have been reported in traditional Mei yu [10] and fermented tilapia surimi [28]. The rapid decline in *Cyanobacteria* during the first 12 h may reflect its poor adaptation to the low-oxygen fermentation environment [34].

At the genus level, marked microbial succession occurred during fermentation. The relative abundance of *Weissella* increased significantly from 6 to 12 h, reaching a maximum of 58.17% at 12 h (*p* < 0.05), followed by a gradual decline to 49.30% at 30 h (*p* < 0.05). *Lactobacillus* increased from 3.59% at 6 h to 20.78% at 12 h (*p* < 0.05) and remained relatively stable at 23.93–25.08% thereafter (*p* > 0.05), while *Lactococcus* increased significantly to 6.67% at 30 h (*p* < 0.05). These results showed that a LAB-dominated microbial community developed during the subsequent fermentation of SFCCJ-pretreated fillets. *Weissella* is widely detected in fermented plant and fish products and may improve their nutritional and sensory properties [65]. Consistent with the present study, it commonly predominates during the early stages of fermentation and is subsequently partially replaced by *Lactobacillus* [66]. Consistent with our results, *Lactobacillus* has also been reported as a major bacterial genus in Hainan yucha [67] and fermented tilapia surimi [1]. In contrast, *Chroococcus*, *Plectonema*, *Bacillus*, *Mycobacterium*, *Methylobacterium*-*Methylorubrum*, *Bradyrhizobium*, and *Sphingomonas* declined to below 1% after 12 h (*p* < 0.05), suggesting suppression as LAB became dominant. Similar inhibition of non-LAB populations by LAB dominance has been reported in fermented mackerel surimi [68]. Meanwhile, *Pantoea*, *Enterobacter*, and *Citrobacter* remained detectable or increased in relative abundance during later fermentation, suggesting their persistence under the fermentation conditions. These genera may therefore be associated with flavor and other quality changes during fermentation.

### 3.5. Potential Functional Microbiota Associated with Quality Development Based on Microbial Metabolic Network Analysis

**Figure 6:**
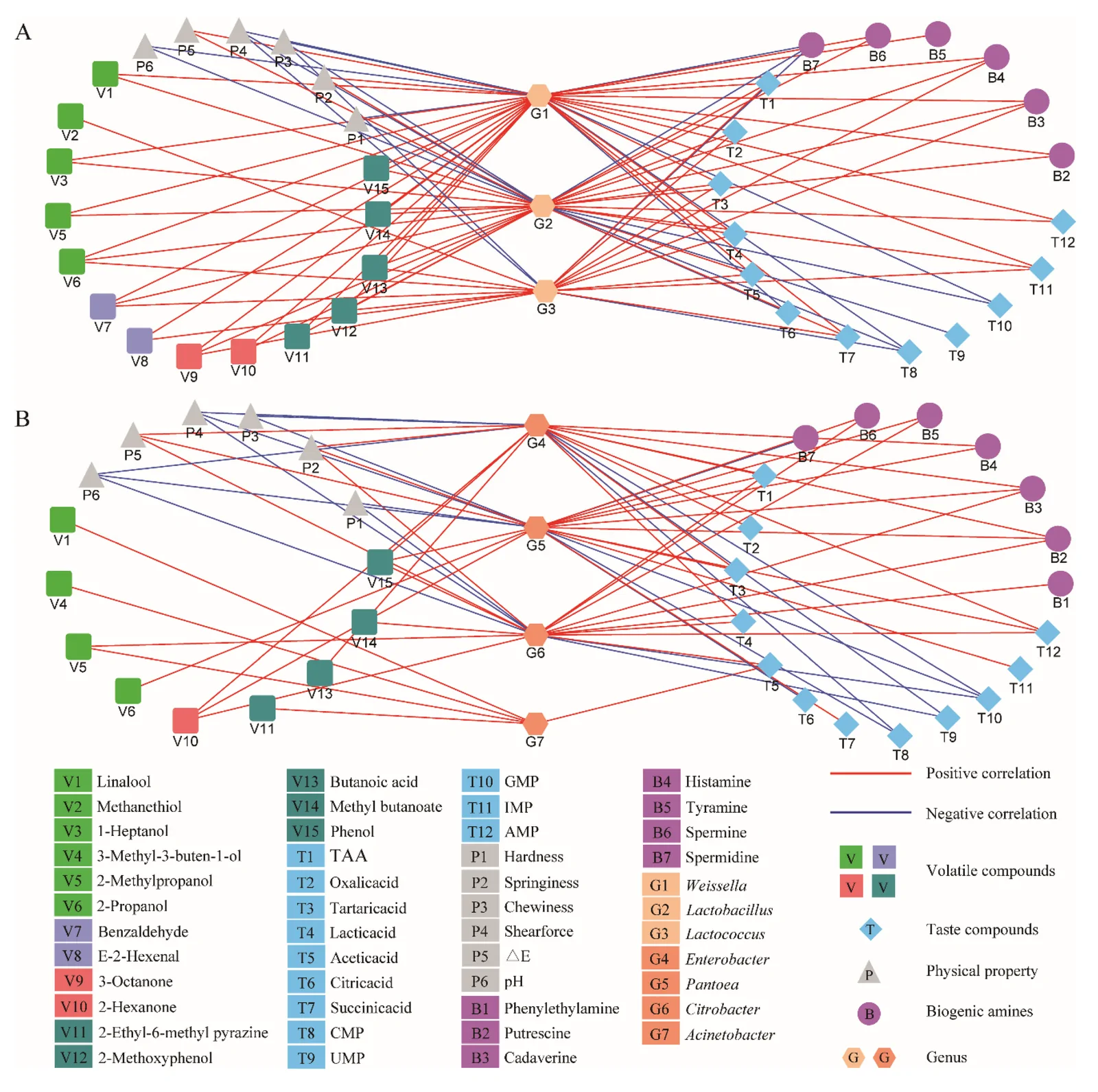
Microbial metabolic network based on correlations between bacterial genera and core metabolites. (**A**) Correlation network of *Weissella*, *Lactobacillus*, and *Lactococcus* (G1–G3). (**B**) Correlation network of *Enterobacter*, *Pantoea*, *Citrobacter*, and *Acinetobacter* (G4–G7). The hexagons represent the main genera, the squares represent the characteristic volatile compounds, and the diamonds, triangles, and circles represent major taste compounds, physicochemical properties, and biogenic amines, respectively. The red and blue lines represent positive and negative correlations, respectively, between the genera and core metabolites.

As fermentation is a process based on microbial metabolism, microorganisms play important roles in quality development and the formation of characteristic fermentation flavors [61]. Potential functional microbiota are generally characterized by relatively high abundance and close associations with key fermentation processes or metabolites [67]. As illustrated in Figure 6, a microbial metabolic network was constructed based on Pearson correlations between bacterial genera and core metabolites. Genera with relative abundance > 1% and significant correlations with these variables (|r| > 0.60, FDR-adjusted *p* < 0.05, Table S8) were considered the principal potential functional microorganisms associated with quality and characteristic flavor development during tilapia fillet fermentation.

*Weissella* was identified as a principal potential functional microorganism associated with quality and flavor development during tilapia fillet fermentation. It was significantly correlated with 24 flavor metabolites and 6 physicochemical properties. Among the volatile compounds, *Weissella* showed positive correlations with several alcohols, aldehydes, ketones, acids, esters, pyrazines, and phenols, including linalool, benzaldehyde, 3-octanone, butanoic acid, methyl butanoate, 2-ethyl-6-methylpyrazine, and phenol (r = 0.844−0.923). It was also positively correlated with T-AA (r = 0.952), lactic acid (r = 0.932), acetic acid (r = 0.841), IMP (r = 0.817), and AMP (r = 0.881), but negatively correlated with citric acid (r = −0.889). In addition, *Weissella* was positively correlated with ΔE and springiness, but negatively correlated with hardness, chewiness, shear force, and pH (|r| = 0.830−0.970). Consistent with the present results, Nazani et al. [69] and Wang et al. [11] reported that *Weissella* promoted the production of organic acids, including lactic and acetic acids, thereby improving the texture and flavor of fermented fish sauce and Suanzuoyu. Zhong et al. [70] identified *Weissella* as a core microorganism in traditionally fermented sour meat, with positive associations with several esters, alcohols, and organic acids. These results suggest that *Weissella* may have been involved in acidification, texture changes, and the accumulation of characteristic flavor metabolites during tilapia fillet fermentation.

*Lactobacillus* was also identified as a principal potential functional microorganism during tilapia fillet fermentation. It was significantly correlated with 12 volatile components, 12 taste components, and 6 physicochemical properties. Specifically, it was positively correlated with several characteristic aroma compounds, including linalool, 1-heptanol, benzaldehyde, 3-octanone, butanoic acid, methyl butanoate, 2-ethyl-6-methylpyrazine, and 2-methoxyphenol (r = 0.914−0.956). *Lactobacillus* was also positively correlated with T-AA (r = 0.990), lactic acid (r = 0.910), tartaric acid (r = 0.942), IMP (r = 0.867) and AMP (r = 0.909), but negatively correlated with citric acid (r = −0.880). In addition, it was positively correlated with springiness and ΔE and negatively associated with hardness, chewiness, shear force, and pH (|r| = 0.821−0.997). Many *Lactobacillus* starter cultures, particularly *L. plantarum* and *L. sakei*, are widely used to improve the flavor and quality of fermented fish products [9]. Zhang et al. [44] reported that *Lactobacillus* increased desirable aroma compounds while reducing fishy volatiles in low-salt fermented tilapia surimi. In fermented plant products, it has also been correlated with fruity aroma formation, organic acid accumulation, and reduced hardness [71,72]. In this study, the abundance of *Lactobacillus* increased after SFCCJ pretreatment and was closely associated with acidification, texture changes, and the accumulation of characteristic flavor metabolites during subsequent fermentation.

*Lactococcus* also showed significant correlations with 16 flavor metabolites, including 8 volatile compounds (e.g., 2-propanol, E-2-hexenal, 3-octanone, 2-methoxyphenol, butanoic acid) and 8 taste components (e.g., T-AA, lactic acid, succinic acid, IMP), as well as chewiness and shear force. Previous studies have identified *Lactococcus* as a dominant genus in dry-fermented sausages and reported positive associations with the formation of esters, aldehydes, and ketones [61]. Inoculation with *L. lactis* M10 together with *Weissella cibaria* M3 has also been shown to promote characteristic volatile formation in stinky mandarin fish [73]. These findings suggest that *Lactococcus* may also be involved in characteristic flavor and textural changes during fermentation. Although *Weissella*, *Lactobacillus*, and *Lactococcus* are generally regarded as relatively safe LAB and may inhibit BA formation in fermented foods [74], some *Lactobacillus* and *Lactococcus* strains possess amino acid decarboxylase activity [75]. In the present study, these genera were positively correlated with cadaverine and histamine.

*Enterobacter*, *Pantoea* and *Citrobacter* were also identified as principal potential functional microorganisms associated with flavor metabolite accumulation during tilapia fillet fermentation. All three genera were positively correlated with several characteristic volatiles, including 2-hexanone, methyl butanoate, and phenol, as well as T-AA and AMP, but negatively correlated with GMP. Similar associations with flavor metabolite formation have been reported for *Enterobacter* in pickled radish [76] and *Citrobacter* in fermented tilapia surimi [1]. Notably, these genera were also positively correlated with several BAs. Putrescine and cadaverine were correlated with all three genera, while histamine was correlated with *Pantoea* and *Enterobacter* and tyramine with *Pantoea* and *Citrobacter*. Given the amino acid decarboxylase activity reported for many species within these genera [77], they may have been involved in BA formation during fermentation. In contrast, *Acinetobacter* and other low-abundance genera showed fewer correlations with flavor metabolites and were significantly suppressed during fermentation, suggesting a limited contribution to overall quality development.

## 4. Conclusions

This study demonstrated that Chinese cabbage juice marination pretreatment altered the initial substrate and microbial characteristics of tilapia fillets and was followed by marked quality and microbial community structure changes during subsequent fermentation. SFCCJ pretreatment enriched fermentable substrates and functional components in tilapia fillets; increased the abundances of *Pantoea*, *Weissella*, and *Lactobacillus*; and improved the inter-sample consistency of the initial microbial community. During subsequent fermentation of the pretreated fillets, lightness and redness increased, while hardness, chewiness, and shear force decreased and springiness increased. Meanwhile, FAAs, 5′-nucleotides and organic acids accumulated; characteristic volatiles associated with floral, green, and fruity odors increased (especially alcohols, aldehydes, and ketones) and fishy odor-related compounds (e.g., 2-ethyl-1-hexanol, E-2-octenal, and diallyl disulfide) decreased. A LAB-dominated microbial community, mainly comprising *Weissella*, *Lactobacillus*, and *Lactococcus*, developed from 12 h onward. Microbial metabolic network analysis showed that *Weissella* and *Lactobacillus* were closely associated with acidification, texture changes, and characteristic flavor metabolite accumulation, while *Lactococcus* showed similar associations. In contrast, *Pantoea*, *Enterobacter*, and *Citrobacter* were associated with BA accumulation, indicating their potential relevance to fermentation safety. This study provides a promising strategy for the targeted quality improvement of tilapia and a basis for the further isolation, screening, and validation of potentially functional microorganisms suitable for tilapia fermentation. Furthermore, future studies should compare different vegetable-derived marinades to further evaluate their effects on the initial substrate composition, microbial community, and subsequent fermentation quality of tilapia fillets.

## Figures and Tables

**Table 1:** Changes in pH, TVC, LAB, color, TPA, and shear force of tilapia fillets during fermentation.

Index	Group	Fermentation Time (h)					
		0 h	6 h	12 h	18 h	24 h	30 h
Hardness (N)	SFCCJ	23.15 ± 2.14 ^bc^	25.65 ± 3.57 ^c^	24.22 ± 3.28 ^bc^	24.37 ± 2.46 ^bc^	18.57 ± 1.93 ^a^	20.08 ± 2.26 ^ab^
	Control	23.15 ± 2.48 ^a^	25.62 ± 1.58 ^a^	24.50 ± 2.12 ^a^	25.77 ± 2.71 ^a^	26.99 ± 1.79 ^a^*	25.35 ± 1.67 ^a^*
Springiness (mm)	SFCCJ	2.88 ± 0.23 ^a^	3.05 ± 0.19 ^a^	3.08 ± 0.06 ^ab^	3.15 ± 0.28 ^ab^	3.61 ± 0.06 ^c^	3.34 ± 0.11 ^b^
	Control	2.92 ± 0.25 ^a^	3.13 ± 0.17 ^a^	2.96 ± 0.11 ^a^	3.13 ± 0.34 ^a^	3.17 ± 0.17 ^a^*	3.09 ± 0.27 ^a^*
Chewiness (N.mm)	SFCCJ	0.93 ± 0.12 ^d^	0.86 ± 0.18 ^cd^	0.73 ± 0.09 ^c^	0.48 ± 0.03 ^b^	0.25 ± 0.03 ^a^	0.28 ± 0.03 ^a^
	Control	0.95 ± 0.07 ^a^	0.87 ± 0.06 ^ab^	0.75 ± 0.02 ^bc^	0.73 ± 0.03 ^bc^*	0.73 ± 0.06 ^c^*	0.74 ± 0.01 ^bc^*
Shear force (N)	SFCCJ	8.11 ± 1.00 ^c^	7.90 ± 1.31 ^c^	6.14 ± 0.56 ^b^	5.64 ± 0.41 ^ab^	4.54 ± 0.32 ^a^	5.21 ± 0.14 ^ab^
	Control	8.46 ± 0.74 ^b^	8.26 ± 1.08 ^b^	7.71 ± 1.07 ^ab^*	7.13 ± 1.45 ^ab^*	6.99 ± 0.68 ^ab^*	6.60 ± 0.16 ^a^*
L^*^	SFCCJ	25.04 ± 0.95 ^a^	25.21 ± 0.74 ^a^	25.29 ± 0.49 ^a^	27.45 ± 1.18 ^ab^	28.79 ± 1.46 ^bc^	29.94 ± 0.95 ^c^
	Control	25.34 ± 0.98 ^ab^	25.13 ± 0.99 ^ab^	26.72 ± 0.74 ^c^*	25.22 ± 0.86 ^a^*	26.47 ± 0.95 ^bc^*	26.78 ± 0.64 ^c^*
a^*^	SFCCJ	1.15 ± 0.18 ^a^	1.39 ± 0.16 ^b^	1.62 ± 0.03 ^c^	1.87 ± 0.16 ^d^	2.02 ± 0.23 ^de^	2.19 ± 0.08 ^e^
	Control	1.24 ± 0.21 ^a^	1.45 ± 0.40 ^a^	1.32 ± 0.18 ^a^	1.37 ± 0.13 ^a^*	1.47 ± 0.17 ^a^*	1.41 ± 0.12 ^a^*
b^*^	SFCCJ	−0.58 ± 0.12 ^d^	−0.94 ± 0.16 ^cd^	−1.24 ± 0.16 ^c^	−1.64 ± 0.16 ^b^	−2.05 ± 0.43 ^a^	−1.96 ± 0.31 ^ab^
	Control	−0.93 ± 0.75 ^c^	−1.14 ± 0.47 ^c^	−1.79 ± 0.09 ^b^*	−2.79 ± 0.09 ^a^*	−2.93 ± 0.50 ^a^*	−3.32 ± 0.22 ^a^*
△E	SFCCJ	0	0.11 ± 0.01 ^a^	0.07 ± 0.01 ^a^	3.72 ± 0.12 ^b^	8.47 ± 0.36 ^c^	13.51 ± 1.19 ^d^
	Control	0	0.07 ± 0.00 ^a^*	1.48 ± 0.02 ^b^*	2.38 ± 0.05 ^c^*	2.66 ± 0.10 ^c^*	3.91 ± 0.42 ^d^*
pH	SFCCJ	6.71 ± 0.01 ^e^	6.68 ± 0.09 ^e^	6.50 ± 0.02 ^d^	6.30 ± 0.02 ^c^	5.77 ± 0.02 ^b^	5.42 ± 0.02 ^a^
	Control	6.69 ± 0.04 ^c^	6.72 ± 0.02 ^c^	6.69 ± 0.04 ^c^*	6.53 ± 0.03 ^b^*	6.58 ± 0.03 ^b^*	6.35 ± 0.02 ^a^*
TVC (log CFU/g)	SFCCJ	5.75 ± 0.03 ^b^	5.82 ± 0.03 ^b^	5.54 ± 0.14 ^a^	6.76 ± 0.04 ^c^	7.00 ± 0.02 ^d^	7.37 ± 0.00 ^e^
	Control	5.57 ± 0.00 ^a^	5.82 ± 0.04 ^b^	5.95 ± 0.03 ^b^	6.16 ± 0.01 ^c^*	6.82 ± 0.03 ^d^*	7.24 ± 0.02 ^e^*
LAB (log CFU/g)	SFCCJ	4.96 ± 0.03 ^a^	5.43 ± 0.07 ^b^	6.84 ± 0.06 ^c^	7.53 ± 0.02 ^d^	8.74 ± 0.08 ^f^	8.44 ± 0.03 ^e^
	Control	4.56 ± 0.07 ^a^	5.35 ± 0.09 ^b^	5.42 ± 0.04 ^b^*	5.44 ± 0.26 ^b^*	6.00 ± 0.03 ^c^*	6.92 ± 0.01 ^d^*

Different lowercase superscript letters within the same group and row indicate significant differences among fermentation times (*p* < 0.05). * indicates a significant difference between the SFCCJ and control groups at the same fermentation time (*p* < 0.05).

**Table 2:** Changes in BAs during tilapia fillet fermentation (mg/kg).

Biogenic Amine	Group	0 h	6 h	12 h	18 h	24 h	30 h
Tryptamine	SFCCJ	ND	ND	ND	ND	ND	ND
	Control	ND	ND	ND	2.34 ± 0.02 ^a^*	2.31 ± 0.01 ^a^*	2.43 ± 0.01 ^b^*
Phenylethylamine	SFCCJ	ND	ND	ND	ND	ND	2.59 ± 0.14
	Control	ND	ND	ND	ND	2.82 ± 0.08 ^a^*	3.26 ± 0.11 ^b^*
Putrescine	SFCCJ	15.98 ± 0.56 ^a^	18.17 ± 1.00 ^b^	23.70 ± 1.49 ^c^	41.03 ± 2.21 ^d^	61.67 ± 2.53 ^e^	67.01 ± 0.59 ^e^
	Control	16.38 ± 0.62 ^a^	37.84 ± 2.83 ^b^*	68.63 ± 1.12 ^c^*	82.00 ± 2.23 ^d^*	86.17 ± 0.87 ^e^*	87.06 ± 0.25 ^e^*
Cadaverine	SFCCJ	7.46 ± 0.56 ^a^	15.83 ± 0.57 ^b^	36.44 ± 1.68 ^c^	41.65 ± 1.44 ^d^	64.14 ± 1.27 ^e^	71.01 ± 0.59 ^f^
	Control	7.03 ± 0.77 ^a^	17.42 ± 1.17 ^b^	36.56 ± 2.74 ^c^	74.84 ± 2.23 ^d^*	86.86 ± 1.17 ^e^*	93.68 ± 0.61 ^f^*
Histamine	SFCCJ	4.83 ± 0.09 ^a^	6.17 ± 0.31 ^b^	11.67 ± 1.72 ^c^	13.80 ± 0.76 ^cd^	15.09 ± 1.14 ^d^	18.12 ± 0.75 ^e^
	Control	5.02 ± 0.13 ^a^	7.76 ± 1.00 ^b^	12.24 ± 0.50 ^c^	16.17 ± 1.00 ^d^*	19.84 ± 1.07 ^e^*	23.19 ± 1.08 ^f^*
Tyramine	SFCCJ	ND	ND	ND	ND	3.41 ± 0.52 ^a^	8.01 ± 0.06 ^b^
	Control	ND	ND	0.35 ± 0.30 ^a^*	0.75 ± 0.47 ^a^*	4.61 ± 0.40 ^b^*	10.22 ± 0.48^c^*
Spermine	SFCCJ	5.52 ± 0.71 ^a^	5.52 ± 0.66 ^a^	5.58 ± 0.22 ^a^	5.67 ± 0.30 ^a^	6.16 ± 0.11 ^b^	6.14 ± 0.15^b^
	Control	5.30 ± 0.66 ^a^	5.30 ± 0.12 ^a^	5.74 ± 0.40 ^a^	5.70 ± 0.27 ^a^	6.00 ± 0.19 ^b^	7.12 ± 0.19^c^*
Spermidine	SFCCJ	9.46 ± 0.54 ^c^	6.44 ± 0.32 ^b^	2.97 ± 0.11 ^a^	ND	ND	ND
	Control	9.23 ± 0.23 ^d^	8.46 ± 0.59 ^d^*	7.07 ± 0.13 ^c^*	4.14 ± 0.25 ^b^*	1.83 ± 0.40 ^a^*	ND
Total biogenic amines	SFCCJ	43.24 ± 2.45 ^a^	52.12 ± 0.22 ^b^	80.34 ± 1.34 ^c^	102.14 ± 2.60 ^d^	150.46 ± 4.53 ^e^	172.86 ± 0.35^f^
	Control	42.96 ± 3.33 ^a^	76.77 ± 2.28 ^b^*	130.58 ± 1.29 ^c^*	186.00 ± 3.00 ^d^*	210.42 ± 1.26 ^e^*	226.94 ± 1.77 ^f^*

Different lowercase superscript letters within the same group and row indicate significant differences among fermentation times (*p* < 0.05). * indicates a significant difference between the SFCCJ and control groups at the same fermentation time (*p* < 0.05).

## Data Availability

The original contributions presented in this study are included in the article/Supplementary Material. Further inquiries can be directed to the corresponding author.

## Supplementary Materials

The following supporting information can be downloaded at: https://www.mdpi.com/article/10.3390/foods15213807/s1. Figure S1: Growth curve of lactic acid bacteria during the spontaneous fermentation of Chinese cabbage juice (expressed as colony counts); Figure S2: Changes in key substrate components and microbial counts during spontaneous fermentation of Chinese cabbage juice and marination of tilapia fillets; Figure S3: PCA plot of microbial community structure in tilapia fillets before (BM) and after (AM) 30 min marination with 6 h spontaneously fermented Chinese cabbage juice; Table S1: Sensory evaluation criteria for fermented tilapia fillets; Table S2: Effects of Chinese cabbage juice fermentation duration on the texture, pH, LAB, and Enterobacteriaceae of 18 h fermented tilapia fillets; Table S3: Relative abundance of bacterial phyla in 6 h spontaneously fermented Chinese cabbage juice and in tilapia fillets before and after 30 min marination; Table S4: Relative abundance of bacterial genus in 6 h spontaneously fermented Chinese cabbage juice and in tilapia fillets before and after 30 min marination; Table S5: Volatile flavor compounds in tilapia fillets during fermentation; Table S6: Characteristic volatile flavor compounds in tilapia fillets during fermentation (VIP > 1, -log_10_ *p* > 3); Table S7: Changes in the peak intensities of 57 volatile flavor compounds during tilapia fillet fermentation; Table S8: Pearson correlation coefficients between bacterial genera and key quality-related variables during tilapia fillet fermentation.
