# Supplementary material for: Effects of Chinese Cabbage Juice Marination Pretreatment on the Quality and Microbial Succession of Fermented Tilapia Fillets
Source: Foods. 2026 Oct 23;15(21):3807. doi: 10.3390/foods15213807 (PMC13763389; doi:10.3390/foods15213807)
Supplement: Supplementary file 1 [file foods-15-03807-s001.zip › foods-4555580-supplementary.pdf]

Supplementary S1

Figure S1 Growth curve of lactic acid bacteria during the spontaneous fermentation of Chinese cabbage juice (expressed as colony counts)

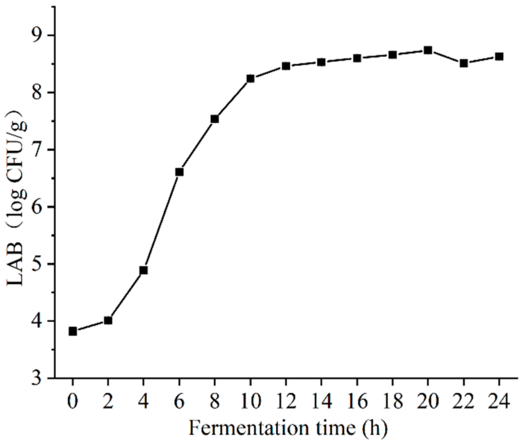

Figure S2 Changes in key substrate components and microbial counts during spontaneous fermentation of Chinese cabbage juice and marination of tilapia fillets

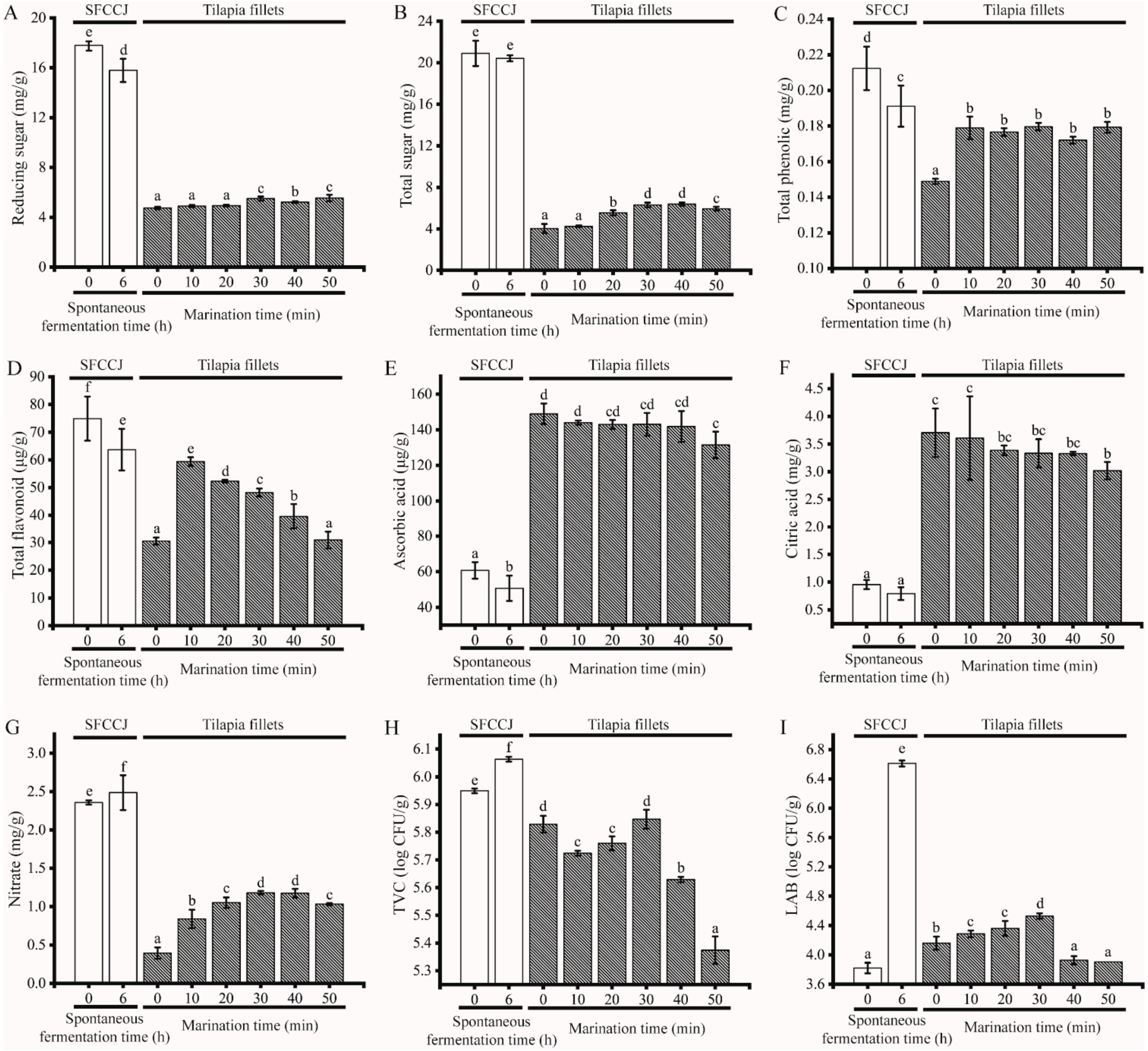

Figure S3 PCA plot of microbial community structure in tilapia fillets before (BM) and after (AM) 30 min marination with 6 h spontaneously fermented Chinese cabbage juice

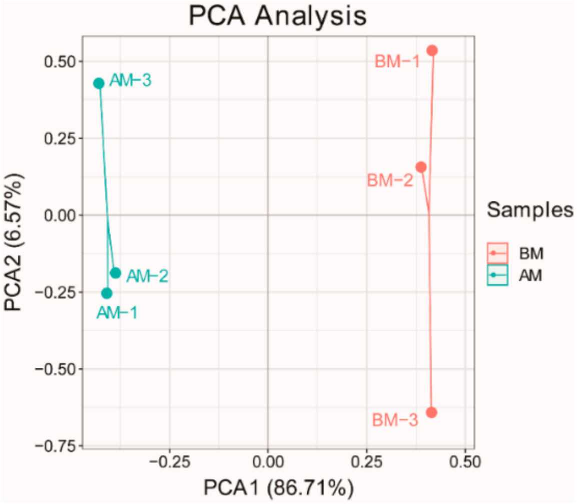

Supplementary S2

Table S1 Sensory evaluation criteria for fermented tilapia fillets.

Attribute	Score		
	**7-9**	**4-6**	**1-3**
Color	Glossy	Slightly glossy	Dull, dark yellow
Aroma	Pleasant vegetable and fermented aroma; no off-odor	Weak characteristic aroma; slight off-odor	Characteristic aroma absent; pronounced off-odor
Taste	Pronounced umami and slight sweetness, mild acidic note	Weak umami and sweetness, noticeable acidic note	No obvious umami or sweetness, strong acidic note
Texture	Tender, springy	Moderately firm, slightly springy	Hard, lacking springiness
Overall acceptability	Highly acceptable	Moderately acceptable	Unacceptable

Table S2 Effects of Chinese cabbage juice fermentation duration on the texture, pH, LAB, and Enterobacteriaceae of 18 h -fermented tilapia fillets.

Index	Fresh tilapia fillets	2% NaCl solution	Fresh juice	6 h -fermented SFCCJ	12 h -fermented SFCCJ
Hardness (N)	23.52±0.93^{b}	25.27±0.90^{c}	23.36±0.68^{b}	21.27±1.00^{a}	23.36±0.97^{b}
Springiness (mm)	2.88±0.12^{a}	3.13±0.14^{bc}	3.31±0.19^{c}	3.61±0.06^{d}	3.01±0.13^{ab}
Chewiness (N.mm)	0.97±0.15^{c}	0.73±0.02^{b}	0.67±0.02^{b}	0.48±0.03^{a}	0.68±0.03^{b}
Shear force (N)	8.59±1.99^{b}	7.71±2.13^{ab}	6.14±0.56^{a}	5.64±0.41^{a}	7.13±1.45^{ab}
pH	6.78±0.03^{e}	6.56±0.03^{d}	6.30±0.02^{c}	5.82±0.03^{b}	5.48±0.04^{a}
LAB (log CFU/g)	4.16±0.10^{a}	4.87±0.25^{b}	6.42±0.07^{c}	7.07±0.13^{d}	7.98±0.14^{e}
Enterobacteriaceae (log CFU/g)	4.33±0.11^{a}	4.52±0.06^{b}	4.79±0.03^{c}	4.74±0.04^{c}	5.60±0.03^{d}

Table S3 Relative abundance of bacterial phyla in 6 h spontaneously fermented Chinese cabbage juice and in tilapia fillets before and after 30 min marination.

Bacterial phyla	SFCCJ	BM	AM	Abundance change
Firmicutes	7.33±0.89%	2.79±0.09%	5.45±0.11%	↑**
Proteobacteria	88.55±0.43%	25.37±1.12%	36.58±0.32%	↑**
Cyanobacteria	ND	60.01±0.00%	52.79±0.36%	↓**
Actinobacteriota	2.31±0.21%	9.56±1.68%	3.39±0.04%	↓**
Bacteroidota	1.08±0.24%	0.98±0.05%	0.76±0.09%	↓*
Myxococcota	0.47±0.06%	0.82±0.05%	0.99±0.05%	↑**
Fusobacteriota	ND	0.40±0.39%	0.01±0.00%	↓
Bdellovibrionota	0.21±0.02%	0.00±0.00%	0.01±0.01%	↑
Others	0.04±0.03%	0.07±0.02%	0.03±0.01%	↓*

* and ** indicate significant differences at $p < 0.05$ and $p < 0.01$, respectively.

Table S4 Relative abundance of bacterial genus in 6 h spontaneously fermented Chinese cabbage juice and in tilapia fillets before and after 30 min marination.

**Bacterial *genus***	**SFCCJ**	**BM**	**AM**	**Abundance change**	**Bacterial phyla**
Plectonema	ND	26.37±1.51%	22.71±0.59%	↓*	*Cyanobacteria*
Chroococcus	ND	37.11±0.97%	31.28±0.42%	↓**	*Cyanobacteria*
Renibacterium	ND	7.92±0.37%	1.41±0.11%	↓**	*Actinobacteriota*
Burkholderia-Caballeronia-Paraburkholderia	2.07±0.12%	5.43±0.08%	5.00±0.13%	↓**	*Proteobacteria*
Bradyrhizobium	2.66±0.20%	2.93±0.12%	2.34±0.09%	↓**	*Proteobacteria*
Mycobacterium	0.96±0.20%	2.04±0.17%	2.06±0.09%	↑	*Actinobacteriota*
Stenotrophomonas	0.19±0.03%	1.57±0.07%	0.25±0.04%	↓**	*Proteobacteria*
Methylobacterium-Methylorubrum	0.53±0.11%	2.33±0.02%	2.32±0.06%	↓	*Proteobacteria*
Sphingomonas	4.75±0.24%	1.54±0.05%	2.07±0.08%	↑**	*Proteobacteria*
Pseudomonas	4.33±0.16%	3.63±0.08%	1.32±0.04%	↓**	*Proteobacteria*
Pajaroellobacter	0.45±0.06%	0.82±0.05%	1.07±0.04%	↑**	*Myxococcota*
Pantoea	42.74±3.13%	0.08±0.00%	1.25±0.17%	↑**	*Proteobacteria*
Weissella	6.95±0.09%	0.19±0.02%	1.35±0.04%	↑**	*Firmicutes*
Lactobacillus	6.66±0.06%	0.02±0.00%	1.18±0.10%	↑**	*Firmicutes*
Paenibacillus	ND	0.73±0.02%	0.43±0.00%	↓**	*Firmicutes*
Ralstonia	1.23±0.24%	0.33±0.03%	0.41±0.03%	↑*	*Proteobacteria*
Enterobacter	2.97±0.12%	0.33±0.04%	0.36±0.05%	↑	*Proteobacteria*
Ruminococcus torques group	ND	0.43±0.02%	0.07±0.02%	↓**	*Firmicutes*
Cetobacterium	ND	0.40±0.39%	0.00±0.00%	↓	*Proteobacteria*
Bacillus	0.60±0.06%	0.11±0.03%	0.26±0.05%	↑*	*Firmicutes*
Others	22.91±1.03%	6.57±1.37%	22.01±0.81%	↑**	/

* and ** indicate significant differences at $p < 0.05$ and $p < 0.01$, respectively.

Table S5 Volatile flavor compounds in tilapia fillets during fermentation.

Category	Count	Compound	CAS#	Formula	MW	RI	Rt [sec]	Dt [RIPrel]	Odor type
Alcohols	1	2-Methylpropanol	C78831	C4H10O	74.1	595.1	95.445	1.368	ethereal
	2	2-Methyl-1-butanol	C137326	C5H12O	88.1	722.6	143.325	1.226	ethereal
	3	2-3-Butanediol	C513859	C4H10O2	90.1	795.3	200.34	1.363	creamy
	4	Z-3-Hexenol	C928961	C6H12O	100.2	866.6	273.315	1.221	green
	5	4-Methyl-1-pentanol	C626891	C6H14O	102.2	866.3	273	1.307	nutty
	6	1-Heptanol	C111706	C7H16O	116.2	985.3	475.125	1.39	green
	7	Furaneol	C3658773	C6H8O3	128.1	1073	668.325	1.602	caramellic
	8	Linalool	C60047178	C10H18O	170.3	1064.6	649.32	1.259	floral
	9	2-Ethyl-1-Hexanol	C104767	C8H18O	130.2	1048.9	613.83	1.427	citrus
	10	Ethanol	C64175	C2H6O	46.1	424.6	57.54	1.063	alcoholic
	11	2-Propanol	C67630	C3H8O	60.1	494.9	73.185	1.202	alcoholic
	12	Methanethiol	C74931	CH4S	48.1	465.2	66.57	1.083	sulfurous
	13	3-Methyl-3-buten-1-ol	C763326	C5H10O	86.1	736.8	152.985	1.26	fruity
	14	Furfuryl alcohol	C98000	C5H6O2	98.1	866.4	273.105	1.379	bready
Aldehydes	15	Propanal	C123386	C3H6O	58.1	482.2	70.35	1.138	ethereal
	16	Isopentanal	C590863	C5H10O	86.1	687.6	123.69	1.193	aldehydic
	17	Citronellal	C106230	C10H18O	154.3	1157.7	862.05	1.161	floral
	18	Pentanal	C110623	C5H10O	86.1	685	122.535	1.423	fermented
	19	Heptanal	C111717	C7H14O	114.2	894.7	310.8	1.339	green
	20	Butanal	C123728	C4H8O	72.1	562.9	88.305	1.097	chocolate
	21	Octanal	C124130	C8H16O	128.2	1000.2	506.625	1.409	aldehydic
	22	Nonanal	C124196	C9H18O	142.2	1099.4	728.805	1.479	aldehydic
	23	E-2-Heptenal	C18829555	C7H12O	112.2	961.6	426.93	1.252	green
	24	E-2-Octenal	C2548870	C8H14O	126.2	1074.2	671.055	1.326	fatty
	25	Hexanal	C66251	C6H12O	100.2	787.2	193.305	1.269	green
	26	E-2-Hexenal	C6728263	C6H10O	98.1	850.4	254.415	1.182	green
	27	Benzaldehyde	C100527	C7H6O	106.1	952.3	408.765	1.143	fruity
	28	Benzene acetaldehyde	C122781	C8H8O	120.2	1063.9	647.64	1.538	green
Ketones	29	2-Hexanone	C591786	C6H12O	100.2	787.1	193.2	1.188	fruity
	30	3-Octanone	C106683	C8H16O	128.2	984.2	472.815	1.308	herbal
	31	2-Pentanone	C107879	C5H10O	86.1	684.3	122.22	1.388	fruity
	32	2-Octanone	C111137	C8H16O	128.2	997.8	501.375	1.326	earthy
	33	2-3-Pentanedione	C600146	C5H8O2	100.1	680.8	120.645	1.22	buttery
	34	2-Propanone	C67641	C3H6O	58.1	467.1	66.99	1.121	solvent
	35	2-Butanone	C78933	C4H8O	72.1	590.8	94.5	1.242	ethereal
	36	3-Pentanone	C96220	C5H10O	86.1	684.1	122.115	1.352	ethereal
	37	2-Heptanone	C110430	C7H14O	114.2	890	303.975	1.262	cheesy
Esters	38	Methyl butanoate	C623427	C5H10O2	102.1	722.9	143.535	1.151	fruity
	39	Bornyl acetate	C76493	C12H20O2	196.3	1284.2	1151.43	1.164	balsamic
	40	Propyl butanoate	C105668	C7H14O2	130.2	893.2	308.49	1.691	fruity
	41	Ethyl acetate	C141786	C4H8O2	88.1	597.9	96.075	1.33	ethereal
	42	Z-3-Hexen-1-ol acetate	C3681718	C8H14O2	142.2	1000.6	507.36	1.812	green
	43	Methyl acetate	C79209	C3H6O2	74.1	528	80.535	1.073	ethereal
	44	Ethyl isobutyrate	C97621	C6H12O2	116.2	736.3	152.67	1.192	fruity
Phenols	45	2-Methoxyphenol	C90051	C7H8O2	124.1	1095.7	720.3	1.238	phenolic
	46	Phenol	C108952	C6H6O	94.1	996.3	498.33	1.065	phenolic

	47	2-Cresol	C95487	C7H8O	108.1	1057.9	633.99	1.105	phenolic
Acids	48	Pentanoic acid	C109524	C5H10O2	102.1	916.8	345.24	1.243	cheesy
	49	Butanoic acid	C107926	C4H8O2	88.1	794.2	199.395	1.172	cheesy
	50	Propionic acid	C79094	C3H6O2	74.1	738.6	154.35	1.109	cheesy
Pyrazines	51	2-Ethyl-6-methyl pyrazine	C13925036	C7H10N2	122.2	986.1	476.7	1.197	potato
	52	2-5-Dimethylpyrazine	C123320	C6H8N2	108.1	916.2	344.19	1.497	chocolate
Sulfides	53	2-Acetylthiazole	C24295032	C5H5NOS	127.2	1019.8	549.15	1.122	popcorn
	54	Diallyl disulfide	C2179579	C6H10S2	146.3	1074	670.635	1.644	alliaceous
Ether	55	Tert-butylmethylether	C1634044	C5H12O	88.1	553.5	86.20499	1.13	minty
	56	1-4-Dioxan	C123911	C4H8O2	88.1	723.1	143.64	1.318	-
Furans	57	2-Ethylfuran	C3208160	C6H8O	96.1	685	122.535	1.295	green

RI, retention index; Rt, retention time; Dt, relative drift time (normalized to the reactant ion peak, RIP).

Table S6 Characteristic volatile flavor compounds in tilapia fillets during fermentation. (VIP>1, $-\log_{10} p>3$)

**Category**	**Count**	**Compound**	**VIP value**	**-Log₁₀ (*p*)**	**BH-FDR -Log₁₀ (*p*)**	**Content change**	**Odor type**
Acids	1	Propionic acid	1.6623	4.8631	4.5549	↑	cheesy
Alcohols	2	Furfuryl alcohol	1.5542	4.2463	4.0315	↓	bready
Aldehydes	3	E-2-Octenal	1.4828	4.2085	3.8933	↓	fatty
Aldehydes	4	Benzaldehyde	1.4800	7.1919	6.7211	↑	fruity
Alcohols	5	Linalool	1.4535	10.226	9.8423	↑	floral
Pyrazines	6	2-Ethyl-6-methyl pyrazine	1.4142	11.288	10.6276	↑	potato
Alcohols	7	Methanethiol	1.4091	4.2751	4.0443	↑	sulfurous
Sulfides	8	Diallyl disulfide	1.3868	3.6544	3.3196	↓	alliaceous
Phenols	9	2-Methoxyphenol	1.3779	8.2499	8.0475	↑	phenolic
Alcohols	10	1-Heptanol	1.3701	11.518	11.0189	↑	green
Aldehydes	11	Benzene acetaldehyde	1.3582	12.319	11.2350	↓	green
Ketones	12	3-Octanone	1.3480	9.9515	9.5622	↑	herbal
Ketones	13	3-Pentanone	1.3395	3.4619	3.1583	↓	ethereal
Alcohols	14	3-Methyl-3-buten-1-ol	1.3386	4.3424	4.1553	↓	fruity
Alcohols	15	4-Methyl-1-pentanol	1.3210	3.8353	3.6647	↓	nutty
Ketone	16	2-Hexanone	1.3080	6.8933	6.5907	↑	fruity
Alcohols	17	Z-3-Hexenol	1.2353	3.3432	3.1317	↓	green
Aldehydes	18	E-2-Hexenal	1.2129	5.4627	4.9708	↑	green
Acids	19	Butanoic acid	1.1211	7.2763	6.8214	↑	cheesy
Esters	20	Methyl butanoate	1.1077	6.8974	6.6492	↑	fruity
Alcohols	21	2-Methylpropanol	1.1047	5.7460	5.3209	↑	ethereal
Alcohols	22	2-Ethyl-1-Hexanol	1.0664	5.8564	5.5006	↓	ethereal
Phenols	23	Phenol	1.0468	4.2755	4.0490	↑	phenolic
Alcohols	24	2-Propanol	1.0355	7.2165	6.8204	↑	alcoholic

Table S7 Changes in the peak intensities of 57 volatile flavor compounds during tilapia fillet fermentation.

Category	Count	Compound	Odor type	0h	6h	12h	18h	24h	30h
Alcohols	1	2-Methylpropanol	ethereal	24.71433	59.48133	81.72433	102.848	297.9667	330.6407
	2	2-Methyl-1-butanol	ethereal	124.908	116.3223	144.8373	276.9657	399.401	328.3053
	3	2-3-Butanediol	creamy	140.1883	81.41967	98.19567	79.49767	348.8843	579.3573
	4	Z-3-Hexenol	green	299.034	184.7927	314.5093	182.8187	139.745	126.3693
	5	4-Methyl-1-pentanol	nutty	348.8753	205.3443	361.11	228.307	149.8053	145.9037
	6	1-Heptanol	green	448.145	8585.625	8648.045	7789.861	8348.851	8048.413
	7	Furaneol	caramellic	111.8377	228.0627	419.6503	321.117	298.793	253.372
	8	Linalool	floral	815.2903	4972.054	4551.717	4829.046	4787.695	4842.984
	9	2-Ethyl-1-Hexanol	citrus	7444.158	2416.859	4860.411	1472.619	357.1157	362.3707
	10	Ethanol	alcoholic	387.834	367.833	466.4207	562.477	483.279	507.5383
	11	2-Propanol	alcoholic	211.829	202.3757	201.6947	206.427	506.1147	799.3033
	12	Methanethiol	sulfurous	1264.606	1368.167	1466.432	1359.88	1383.819	1776.123
	13	3-Methyl-3-buten-1-ol	fruity	437.3283	213.4887	1063.265	366.753	256.4823	267.5937
	14	Furfuryl alcohol	bready	293.953	121.7893	284.419	142.919	102.894	90.58267
Aldehydes	15	Propanal	ethereal	2104.55	1224.766	1364.383	426.5207	188.0727	198.167
	16	Isopentanal	aldehydic	528.763	596.3103	385.3743	315.5627	225.7263	242.9323
	17	Citronellal	floral	4752.914	2674.451	2151.308	1249.594	876.8993	896.514
	18	Pentanal	fermented	2358.101	796.981	965.279	156.9023	68.568	74.443
	19	Heptanal	green	2897.299	2097.685	2555.591	878.1727	675.0317	602.876
	20	Butanal	chocolate	99.78133	118.6147	188.911	510.5713	759.32	694.636
	21	Octanal	aldehydic	2400.049	1457.645	1107.779	456.8463	413.389	432.6453
	22	Nonanal	aldehydic	2881.186	2370.576	2149.734	1169.086	1065.839	1283.074
	23	E-2-Heptenal	green	2698.405	2139.926	2297.634	1558.25	1064.217	1030.126
	24	E-2-Octenal	fatty	2266.598	1223.371	2923.32	2249.78	1507.616	1467.081
	25	Hexanal	green	2230.239	1204.093	1193.968	203.856	152.318	149.8473
	26	E-2-Hexenal	green	1396.458	1427.108	1542.299	1594.799	1552.973	2148.436
	27	Benzaldehyde	fruity	471.629	11754.14	11977.16	11002.03	11665.71	11338.79
	28	Benzene acetaldehyde	green	571.285	110.1357	445.634	103.2297	73.314	82.38633
Ketones	29	2-Hexanone	fruity	43.97633	212.983	278.6603	277.4463	395.855	425.029
	30	3-Octanone	herbal	40.96167	341.9077	353.0393	389.0537	413.2863	352.081
	31	2-Pentanone	fruity	1540.071	533.5377	1192.499	318.7573	124.1747	142.9543
	32	2-Octanone	earthy	1021.567	1455.564	1992.86	7092.25	3894.883	5912.538
	33	2-3-Pentanedione	buttery	269.0613	378.761	512.2323	988.037	698.9593	699.0337
	34	2-Propanone	solvent	413.997	253.9887	321.75	208.2047	235.1793	407.0267
	35	2-Butanone	ethereal	1326.385	1070.163	1166.686	1113.929	613.39	663.2033
	36	3-Pentanone	ethereal	1361.795	459.576	1526.586	587.8037	267.3373	271.3477
	37	2-Heptanone	cheesy	771.9727	365.781	492.6093	402.1953	378.068	388.673
Esters	38	Methyl butanoate	fruity	83.53767	138.548	147.104	251.4967	348.0627	332.045
	39	Bornyl acetate	balsamic	920.814	453.7477	417.0367	381.937	449.25	576.2713
	40	Propyl butanoate	fruity	1568.6	591.6227	968.9223	196.3927	122.1573	118.2027
	41	Ethyl acetate	ethereal	146.3817	245.0903	352.8473	429.7963	795.2503	904.6413
	42	Z-3-Hexen-1-ol acetate	green	564.2123	270.8193	160.2783	117.8003	127.0083	106.1617
	43	Methyl acetate	ethereal	557.796	216.8087	230.243	229.4287	222.4823	204.4287
	44	Ethyl isobutyrate	fruity	296.208	141.107	460.8337	412.6967	343.2017	310.4773
Phenols	45	2-Methoxyphenol	phenolic	158.3163	718.9763	718.171	858.8627	925.2927	845.5983
	46	Phenol	phenolic	1209.035	1638.356	1981.235	2627.352	2517.689	2625.423
	47	2-Cresol	phenolic	342.3203	125.265	129.103	120.834	119.575	123.5417

Acids	48	Pentanoic acid	cheesy	674.33	385.7217	1235.06	3235.007	3267.983	3189.369
	49	Butanoic acid	cheesy	171.9563	342.8093	385.42	690.224	1535.421	2001.71
	50	Propionic acid	cheesy	591.9103	797.669	799.4717	1046.238	1275.677	1772.524
Pyrazines	51	2-Ethyl-6-methyl pyrazine	potato	476.3417	4345.09	4183.912	4046.276	3975.28	3997.162
	52	2-5-Dimethylpyrazine	chocolate	265.115	261.191	398.2	2512.965	4533.062	5233.298
Sulfurs	53	2-Acetylthiazole	popcorn	137.1037	335.1427	762.5417	548.0713	455.6423	317.4907
	54	Diallyl disulfide	alliaceous	425.3673	184.087	1068.541	549.7257	263.1477	231.4563
Ether	55	Tert-butylmethylether	minty	624.9137	525.5937	589.961	234.332	179.6213	190.6837
	56	1-4-Dioxan	-	37.081	31.01033	35.02667	81.63967	183.8503	152.1917
Furans	57	2-Ethylfuran	green	399.692	672.5143	548.5493	238.8847	145.1927	191.628

Table S8 Pearson correlation coefficients between bacterial genera and key quality-related variables during tilapia fillet fermentation.

	Weissella	*Lactobacillus*	*Lactococcus*	*Enterobacter*	*Pantoea*	*Citrobacter*	*Acinetobacter*
Linalool	0.851*	0.935*	0.339	0.629	0.513	-0.735	0.918*
Methanethiol	0.745	0.726	0.843*	0.662	0.687	0.550	0.276
1-Heptanol	0.853*	0.936*	0.316	0.622	0.460	0.736	0.467
3-Methyl-3-buten-1-ol	-0.156	-0.085	-0.250	0.078	-0.265	0.117	0.889*
2-Methylpropanol	0.894*	0.917*	0.952*	-0.015	0.720	0.929*	0.822*
2-Propanol	0.850*	0.811*	0.985*	0.673	0.835*	0.537	-0.105
Benzaldehyde	0.867*	0.945*	0.816*	0.641	0.483	0.331	0.465
E-2-Hexenal	0.883*	0.786	0.869*	0.697	0.796	0.606	0.104
3-Octanone	0.902*	0.956*	0.863*	0.720	0.592	0.356	0.386
2-Hexanone	0.876*	0.925*	0.797	0.923*	0.891*	0.871*	0.304
2-Ethyl-6-methyl pyrazine	0.844*	0.932*	0.279	0.592	0.439	0.732	0.914*
2-Methoxyphenol	0.906*	0.947*	0.872*	0.784	0.705	0.474	0.299
Butanoic acid	0.923*	0.914*	0.959*	0.943*	0.809	0.710	-0.061
Methyl butanoate	0.900*	0.927*	0.803	0.873*	0.964*	0.834*	-0.025
Phenol	0.890*	0.901*	0.627	0.919*	0.916*	0.942*	0.215
TAA	0.952*	0.990*	0.827*	0.967*	0.981*	0.932*	0.218
Oxalicacid	0.904*	0.903*	0.963*	0.926*	0.797	0.691	-0.057
Tartaricacid	0.912*	0.942*	0.97*	0.863*	0.923*	0.756	0.131
Lacticacid	0.932*	0.910*	0.845*	0.868*	0.582	0.923*	0.164
Aceticacid	0.841*	0.841*	0.816*	0.751	0.437	0.903*	0.875*
Citricacid	-0.889*	-0.880*	-0.768	-0.800	-0.955*	-0.774	0.173
Succinicacid	0.891*	0.900*	0.963*	0.788	0.920*	0.680	-0.062
CMP	-0.934*	-0.959*	-0.937*	-0.885*	-0.952*	-0.800	-0.117
UMP	-0.780	-0.826*	-0.519	-0.910*	-0.766	-0.935*	-0.550
GMP	-0.872*	-0.888*	-0.632	-0.893*	-0.917*	-0.910*	-0.128
IMP	0.817*	0.867*	0.849*	0.776	0.901*	0.699	-0.127
AMP	0.881*	0.909*	0.668	0.913*	0.927*	0.919*	0.153
Hardness	-0.830*	-0.821*	-0.556	-0.684	-0.841*	-0.965*	0.109
Springiness	0.904*	0.922*	0.734	0.833*	0.933*	0.947*	0.005
Chewiness	-0.929*	-0.957*	-0.883*	-0.914*	-0.983*	-0.806	-0.052
Shearforce	-0.970*	-0.997*	-0.942*	-0.980*	-0.981*	-0.838*	-0.267
△E	0.943*	0.919*	0.765	0.825*	0.976*	0.882*	-0.114
pH	-0.951*	-0.951*	-0.776	-0.862*	-0.971*	-0.949*	-0.008
Phenylethylamine	0.738	0.617	0.733	0.489	0.639	0.833*	-0.040
Putrescine	0.943*	0.948*	0.789	0.868*	0.984*	0.889*	-0.039
Cadaverine	0.963*	0.993*	0.906*	0.961*	0.978*	0.878*	0.224
Histamine	0.979*	0.985*	0.952*	0.978*	0.970*	0.652	0.302
Tyramine	0.848*	0.797	0.678	0.659	0.819*	0.978*	-0.089
Spermine	0.869*	0.902*	0.722	0.800	0.924*	0.922*	-0.068
Spermidine	-0.875*	-0.910*	-0.971*	-0.949*	-0.899*	-0.671	-0.328

Values represent Pearson correlation coefficients (r). * indicates statistical significance after BH-FDR correction (adjusted $p < 0.05$).
